# Ovalbumin-specific CD4^+^ and CD8^+^ T cells contribute to different susceptibility for Theiler’s murine encephalomyelitis virus persistence

**DOI:** 10.3389/fimmu.2023.1194842

**Published:** 2023-05-24

**Authors:** Rouven Wannemacher, Anna Reiß, Karl Rohn, Fred Lühder, Alexander Flügel, Wolfgang Baumgärtner, Kirsten Hülskötter

**Affiliations:** ^1^Department of Pathology, University of Veterinary Medicine Hannover, Foundation, Hannover, Germany; ^2^Center for Systems Neuroscience, University of Veterinary Medicine Hannover, Foundation, Hannover, Germany; ^3^Department of Biometry, Epidemiology and Data Processing, University of Veterinary Medicine Hannover, Foundation, Hannover, Germany; ^4^Institute of Neuroimmunology and Multiple Sclerosis Research, University Medical Center Göttingen, Göttingen, Germany

**Keywords:** central nervous system, OT mice, Theiler’s murine encephalomyelitis virus (TMEV), OVA-specific CD8+ T cells, OVA-specific CD4+ T cells, microgliosis, T cell dependent susceptibility, neuroimmunology

## Abstract

Theiler’s murine encephalomyelitis virus (TMEV) is the causative agent of TMEV-induced demyelinating disease (TMEV-IDD); a well-established animal model for the chronic progressive form of human multiple sclerosis (MS). In susceptible mice with an inadequate immune response, TMEV-IDD is triggered by virus persistence and maintained by a T cell mediated immunopathology. OT-mice are bred on a TMEV-resistant C57BL/6 background and own predominantly chicken ovalbumin (OVA)-specific populations of CD8^+^ T cells (OT-I) or CD4^+^ T cells (OT-II), respectively. It is hypothesized that the lack of antigen specific T cell populations increases susceptibility for a TMEV-infection in OT-mice on a TMEV-resistant C57BL/6 background. OT-I, OT-II, and C57BL/6 control mice were infected intracerebrally with the TMEV-BeAn strain. Mice were scored weekly for clinical disease and after necropsy, histological and immunohistochemical evaluation was performed. OT-I mice started to develop progressive motor dysfunction between 7 and 21 days post infection (dpi), leading up to hind limb paresis and critical weight loss, which resulted in euthanasia for humane reasons between 14 and 35 dpi. OT-I mice displayed a high cerebral virus load, an almost complete absence of CD8^+^ T cells from the central nervous system (CNS) and a significantly diminished CD4^+^ T cell response. Contrarily, only 60% (12 of 20) of infected OT-II mice developed clinical disease characterized by mild ataxia. 25% of clinically affected OT-II mice (3 of 12) made a full recovery. 5 of 12 OT-II mice with clinical disease developed severe motor dysfunction similar to OT-I mice and were euthanized for humane reasons between 13 and 37 dpi. OT-II mice displayed only low virus-immunoreactivity, but clinical disease correlated well with severely reduced infiltration of CD8^+^ T cells and the increased presence of CD4^+^ T cells in the brains of OT-II mice. Though further studies are needed to reveal the underlying pathomechanisms following TMEV infection in OT mice, findings indicate an immunopathological process as a main contributor to clinical disease in OT-II mice, while a direct virus-associated pathology may be the main contributor to clinical disease in TMEV-infected OT-I mice.

## Introduction

In addition to inflammatory changes, neurodegeneration characterized by neuronal loss, axonopathy and myelin loss, is a hallmark of many chronic central nervous system (CNS) virus infections (1–6). Autoimmunity and immunopathology are key factors for the development and progression of most degenerative CNS diseases (1–6). As the main cell populations responsible for antiviral immune response in the CNS, microglia and T cells have both beneficial as well as detrimental effects in CNS disease (1–3, 6–8). Microglia contribute to neurodegeneration and neurocognitive impairment in various diseases including chronic West Nile virus and Zika virus infection (1). Immunopathological mechanisms mediated by proinflammatory T helper cells and their interaction with microglia have additionally been shown to contribute to disease progression as shown for in multiple sclerosis (MS), amyotrophic lateral sclerosis, Parkinson’s disease and Alzheimer’s disease (3, 5, 9–15). In MS, infiltration of cytotoxic, CD8^+^ T cells is also closely linked to demyelination and axonal damage (6). Additionally, in humans, several Picornavirus infections, including Poliovirus, Coxsackievirus and Saffoldvirus cause or contribute to acute and progressive CNS-diseases (16–19). As an established animal model, the intracerebral infection of mice with Theiler’s murine encephalomyelitis virus (TMEV) serves as a valuable tool to study possible underlying mechanisms of pathogenesis and immunopathology of MS as well as Picornavirus infection in the CNS of humans and animals (16, 17, 20).

TMEV is a non-enveloped, single-stranded RNA-virus with positive polarity from the family *Picornaviridae* and the causative agent of Theiler’s murine encephalomyelitis (TME) (21–23). TMEV is divided into the highly neurovirulent George’s disease VII (GDVII) subgroup and the attenuated Theiler’s original (TO) subgroup (24). Of the TO subgroup, the most commonly used strains *in vivo* are the Daniel’s (DA) - and the BeAn8386 (BeAn) strain (21, 25, 26).

Viruses of the GDVII subgroup, like the GDVII strain, cause an acute, fatal polioencephalitis in susceptible mice (26, 27). GDVII is more neurotropic and neurovirulent than viruses of the TO subgroup, causing extensive neuronal damage and a lethal disease within 14 days post infection (dpi) (26, 27). There is no virus persistence although the antiviral immune response during the first week of infection is markedly reduced compared to the low virulent TMEV-DA strain (23, 26–28).

After intracerebral infection, virus strains of the TO subgroup cause an acute, transient, subclinical polioencephalitis, which progresses to a chronic, progressive demyelinating leukoencephalomyelitis in the brain and spinal cord, called TMEV-induced demyelinating disease (TMEV-IDD) in susceptible animals like SJL mice (20, 29). This is due to a virus spread to the spinal cord and a change of tropism from neurons to microglia/macrophages, oligodendrocytes and astrocytes (20). Additionally, the white matter is more prominently affected in the chronic phase of the disease (29). After intracerebral infection with viruses of the TO subgroup, clinical signs in susceptible mice start within one month and consist of progressive neurological deficits like unsteady gait, ataxia and paresis of one or more limbs (30). The disease progression and histological lesions of TMEV-IDD closely resemble those of chronic progressive MS in humans (31). Resistant mouse strains such as C57BL/6 mice clear the CNS after the acute phase of disease and do not develop demyelination (29, 32–35). However, they display virus-induced damage of hippocampal neurons during the acute phase of the disease (34, 36).

Demyelination in susceptible mouse strains is a sequela of various simultaneously occurring factors (37–39). The main contributor is a virus-triggered immunopathological process (38, 40, 41). TMEV-antigen presentation to CD4^+^ and CD8^+^ T cells leads to the release of pro-inflammatory cytokines and the activation of microglia as well as attraction of macrophages, which, in turn, cause damage to both infected and uninfected cells through cytotoxic inflammatory mediators (bystander damage) (37–39). This leads to the phagocytosis and presentation of released myelin components by antigen presenting cells, that may trigger a subsequent, myelin-specific autoimmune response of autoreactive T cells (epitope spreading) (40–42). Direct, virus-induced lysis of oligodendrocytes contributes to a lesser degree to demyelination (39).

Susceptibility and resistance to TMEV-IDD are genetically determined (35, 43, 44). Mainly responsible are variations in the H2-D complex of the major histocompatibility complex (MHC) I gene and the gene coding for the constant part of the β-chain of the T cell receptor (TCR) (43, 45). Resistant mice clear the virus from the CNS *via* an early, mainly CD8^+^ T cell-mediated immune reaction to the TMEV-capsid-protein VP2, starting about 7 dpi (35, 44, 46, 47). In previous investigations on the initial phase of TMEV-infection until 7 dpi, CD4^+^- as well as CD8^+^ T cell deficient mice on a C57BL/6 background displayed a lack (CD8^-/-^ mice) or reduced numbers (CD4^-/-^ mice) of VP2-specific cytotoxic T cells, respectively (48). However, progression and immunological alterations at later stages, beyond 7 dpi were not investigated, and thus, a possible susceptibility for a chronic TMEV-infection of these mice was not elucidated (48). It is mentioned that mice on a C57BL/6 background, which lack a CD8^+^ T cell response, develop demyelination after infection with TMEV-DA (49–52). However, despite developing demyelination and virus persistence, these mice displayed preservation of axons and did not develop neurological deficits (49–52). In addition, the effects of functional, but largely unresponsive T cell populations and the resulting unspecific T cell response on the clinical disease and pathomorphology of TME have yet to be investigated.

OT-mice, bred on a C57BL/6 background (53, 54), carry a transgenic, chicken ovalbumin (OVA)-specific TCR (53–55). OT-I mice express a MHC-I restricted TCR, specific for the OVA-derived peptide 257-264 resulting in a preferential thymic selection of OVA-antigen-specific, cytotoxic CD8^+^ T cells (54). While the total number of T cells is not reduced, OT-I mice have roughly 10 times more CD8^+^ T cells than CD4^+^ T cells in the periphery, and in the latter population the transgenic TCR is also expressed to a certain degree (54). OT-II mice carry a MHC-II restricted TCR, specific for the OVA-derived peptide 323-339 leading to preferential selection of CD4^+^ T helper cells (53, 56). Similarly to OT-I mice, OT-II mice also display a constant total T cell count with a fourfold increase in CD4^+^ T cells (53).

Studies with OT-I mice indicated increased susceptibility to infection with TMEV-DA, leading to hind limb paralysis and death or euthanasia for humane reasons within 12 to 17 dpi (57). As, contrary to TMEV-DA, TMEV-BeAn does not cause seizures in C57BL/6 mice, its effect on OT-I mice may differ from that of TMEV-DA and is yet to be determined (20, 58–60).

In the presented study, OT-I and OT-II mice as well as control C57BL/6 wild type mice were intracerebrally infected with TMEV-BeAn to compare the disease progression and clinical outcome, virus persistence, T cell response, microglia proliferation and -activation, neuronal apoptosis, demyelination and axonal damage in these mouse strains. Because the CD8^+^ T cell response is pivotal for TMEV clearance (35, 43), it was investigated whether a non-TMEV reactive CD8^+^ T cell repertoire (OT-I) or a numerically reduced CD8^+^ T cell repertoire without appropriate CD4^+^ T cell help (OT-II) would lead to increased susceptibility for TMEV-infection, possibly causing virus persistence and demyelination, as it was previously seen in CD8-deficient mice (49–52).

## Materials and methods

### Animals

Nineteen OT-I (12♂ and 7♀), 20 OT-II (8♂ and 12♀) and 17 C57BL/6 - control mice (WT) (10♂ and 7♀) were bred at the Institute for Neuroimmunology and Multiple Sclerosis Research (IMSF) of the University Medical Center Göttingen. Animal experiments were performed at the Department of Pathology, University of Veterinary Medicine Hannover in accordance with the Lower Saxony State Office for Consumer Protection and Food Safety (LAVES) under the permission number 33.8-42502-17/2418. Mice were housed in individually ventilated cages (Tecniplast Deutschland GmbH, Hohenpeißenberg, Germany) with 12 h light and 12 h darkness at 20–24°C and 50%–60% humidity. Food (ssniff Spezialdiäten GmbH, Soest, Germany) and water were provided *ad libitum.*

### Study design

All mice were intracerebrally infected with 5,4x10^5^ plaque forming units (PFU) of the Hannover strain of TMEV-BeAn-1-TiHo at 5 weeks of age under general anaesthesia. For general anaesthesia, mice received a intraperitoneal injection with a combination of Medetomidin (0.0005 mg/g bodyweight) and Ketamin (0.1 mg/g bodyweight), both diluted in saline. Mice were examined weekly for general appearance and posture, behaviour and activity, as well as gait. Further examination consisted of determining body weight and RotaRod performance test (RotaRod Treadmill, TSE Technical & Scientific Equipment, Bad Homburg, Germany) as previously described (61).

Animals were euthanized when they reached either the maximum score in any single category (e.g. paralysis of a limb) or the second highest score in two categories, accompanied by a body weight-loss of 20% or more (**Table 1**).

**Table 1:** Criteria and scoring system applied for the clinical examination.

Criteria	Score				
	0	1	2	3	4
Appearance	Smooth, shiny fur; normal posture	Shaggy, dull, fur; normal posture	Shaggy, dull fur; slightly hunched back	Shaggy fur; arched back; incontinence	n.a.
Activity	Active, curious	Very calm: slightly reduced spontaneous activity, normal induced activity	Apathy: moderately reduced spontaneous activity, slightly reduced induced activity	Stupor: no spontaneous activity, significantly reduced induced activity	n.a.
Gait	Normal movement	Mild spinal ataxia: occasional, mild to moderate gait insecurities (mildly shortened stride, occasional unsteadiness and/or stumbling)	Moderate spinal ataxia: regularly occurring mild to moderate gait insecurity (shortened stride, regularly occurring unsteadiness and stumbling, tail flailing)	Severe spinal ataxia: regularly occurring moderate to severe gait insecurities (regularly occurring unsteadiness, stumbling, delayed rise from supine position)	Paresis/paralysis of one or several limbs, falling over, inability to stand up (recumbency)

Scoring system for clinical examination, n.a., not applicable.

For euthanasia, mice were put under general anaesthesia as described above. Thereafter, they received an additional intraperitoneal injection with undiluted Medetomidin (0.001 mg/g bodyweight) and Ketamin (0.2 mg/g bodyweight). Mice were euthanized and post mortally perfused with phosphate buffered saline (PBS) at the following planned necropsy dates: 7 dpi (OT-I n=5 (4♂, 1♀), OT-II n=5 (3♂, 2♀), WT n=5♂), 14 dpi (OT-I n=6 (3♂, 3♀), OT-II n=5 (2♂, 3♀), WT n=6(2♂, 4♀)), 42 dpi (OT-II n=2 (1♂, 1♀)) and 147 dpi (OT-II n=3♀, WT n=6 (3♂, 3♀)). Eight OT-I and five OT-II mice were euthanized for humane reasons between 13 and 37 dpi (OT-I: 13 dpi n=1♂, 16 dpi n=3 (1♂, 2♀), 20 dpi n=2♂, 21 dpi n=1♂, 35 dpi n=1♀; OT-II: 14 dpi n=1♀, 18 dpi n=1♀, 26 dpi n=2 (1♂, 1♀), 37 dpi n=1♂). The right cerebral- and cerebellar hemisphere, as well as tissue samples from cervical-, thoracic- and lumbar spinal cord were fixed for 24 h in 10% neutral buffered formalin and thereafter embedded in paraffin.

In addition, the left cerebral- and cerebellar hemisphere and tissue samples from cervical-, thoracic- and lumbar spinal cord were placed in Tissue-Tek^®^ O.C.T.™ Compound, deep frozen in liquid nitrogen and stored at -80°C.

Formalin fixed, paraffin embedded (FFPE) and O.C.T.-embedded material was cut into 2 μm thick slices. FFPE tissue sections were mounted on glass slides and stained with hematoxylin and eosin (HE) and Luxol Fast Blue (LFB) (62).

### Immunohistochemistry

Immunohistochemistry was performed on FFPE and O.C.T. frozen tissue samples *via* the avidin–biotin–peroxidase complex (ABC)-method (Vector Laboratories Inc., Burlingame, CA, USA) as previously described (63–67). A list of antibodies and the respective methodology can be found in **Table 2**.

**Table 2:** Antibodies, their targets, source and technical details used for immunohistochemistry.

Antibody	Target	Producer	dilution	Pre-treatment	Secondary antibody
Polyclonal rabbit anti-TMEV	TMEV	(68)	1:2000	–	Goat anti-rabbit 1:200
Rabbit anti-Iba1, polyclonal	Microglia/macrophages	Wako Cat.: 019-19741	1:2000	Microwave, citrate buffer 20 min	Goat anti-rabbit 1:200
Glial fibrillary acidic protein (GFAP), rabbit anti-cow, polyclonal	Astrocytes	DAKO Cat.: Z 0334	1:1000	Microwave, citrate buffer 20 min	Goat anti-rabbit 1:200
Polyclonal rabbit-anti-human CD3	T-lymphocytes	DAKO Cat.: A 0452	1:2000	Microwave, citrate buffer 20 min	Goat anti-rabbit 1:200
Mouse anti-alzheimer precursor protein A4 (β-APP) monoclonal	Axonal damage	Sigma-Aldrich Cat.: MAB348	1:2000	Microwave, citrate buffer 20 min	Goat anti-mouse 1:200
Rabbit anti cleaved caspase 3, polyclonal	Neuronal apoptosis	Cell Signaling Technology, Cat.: Asp175	1:2000	Microwave, citrate buffer 20 min	Goat anti-rabbit 1:200
Rat anti mouse CD4, monoclonal	CD4^+^, MHC-II restricted T cells	Dianova Cat.: DIA-404	1:2000	–	Rabbit anti-rat 1:200
Mouse anti human CD8, monoclonal	CD8^+^, MHC-I restricted T cells	DAKO Cat: M7103	1:1000	–	Rabbit anti-mouse 1:200
Mouse anti neuronal nuclear protein, monoclonal	Neurons	Sigma-Aldrich Cat: MAB377	1:1600	Microwave, citrate buffer 20 min	Goat anti-mouse 1:200

TMEV, Theiler’s murine encephalomyelitis virus; MHC, Major histocompatibility complex.-, means that no pre-treatment was performed.

Immunohistochemistry using primary antibodies directed against CD4^+^- and CD8^+^ T cells was performed on O.C.T.-embedded tissue. For this purpose, frozen sections were cut into 5 μm thick slices and mounted on glass slides. Slides were then thawed at room temperature and placed in 0.45% hydrogen peroxide with phosphate buffered saline (PBS) for 30 minutes to inhibit endogenous peroxidases. Afterwards, slides were placed in cover plates and covered in 120μl of rabbit serum in a 1:5 dilution with PBS for 20 minutes to prevent unspecific binding. Thereafter, each slide was covered over night with 120μl of primary antibody dilution (**Table 2**). After washing with PBS, 120μl of a1:200 dilution of secondary antibody were added (**Table 2**). Staining was then performed *via* the ABC-method with 3’3-diaminobenzidine (DAB) as described before (63–66).

### Histological and immunohistochemical evaluation

**Figure 1:**
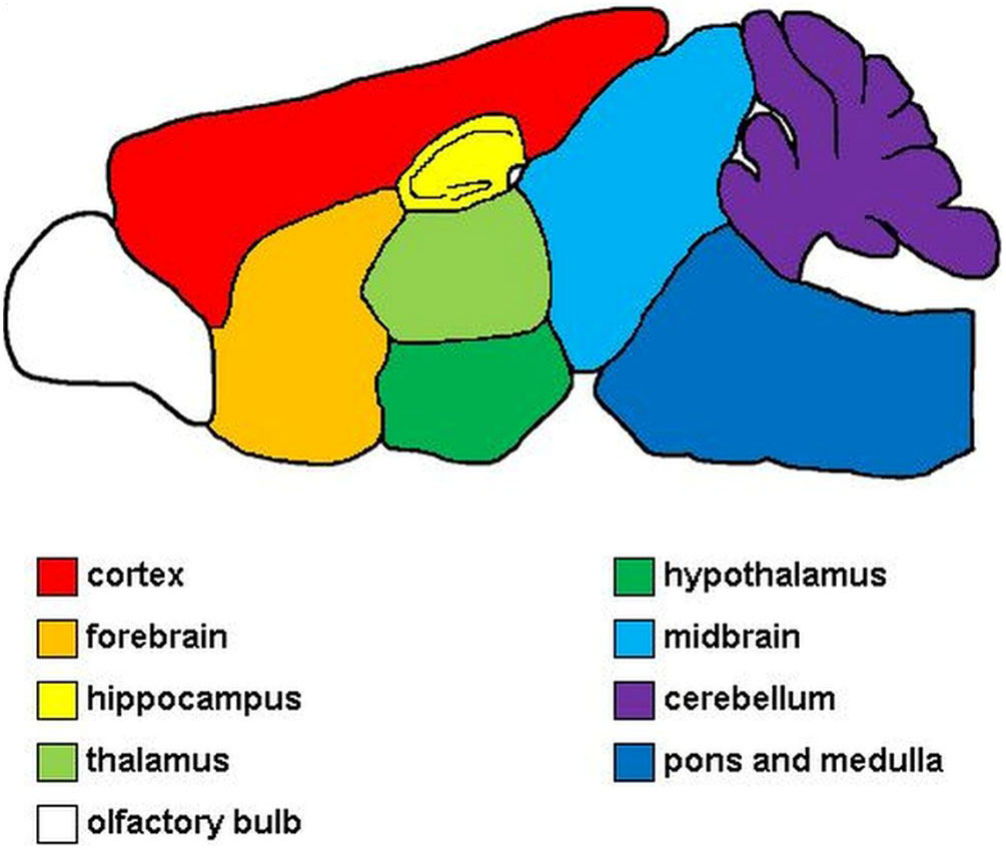
Evaluated areas of the brain. Red, cerebral cortex; orange, forebrain; yellow, hippocampus; light green, thalamus; dark green, hypothalamus; light blue, midbrain; purple, cerebellum; dark blue, pons and medulla; white, olfactory bulb. The olfactory bulb was not included in the evaluation.

Perivascular lymphohistiocytic infiltrates was semi quantitatively evaluated on hematoxylin and eosin (HE) stained longitudinal brain sections and transverse sections of cervical-, thoracic and lumbar spinal cord. The applied scoring systems consisted of four categories (0 = no, 1 = single, 2 = 2-3 layers, 3 = >3 layers of perivascular lymphocytes and or microglia/macrophages, respectively) as described previously (69). In the brain, forebrain, cortex, hippocampus, thalamus, hypothalamus, midbrain, cerebellum, pons and medulla were evaluated separately (**Figure 1**). For evaluation of the whole brain, single area scores were added up into a cumulative total brain score. The olfactory bulb was not included in the evaluation.

For evaluation of immunohistochemical detection of TMEV, CD3, CD4 and CD8 within the brain and spinal cord, positive cells were counted manually for each area of brain and three sections of spinal cord, respectively. The evaluation of immunohistochemical detection of cleaved caspase 3 was performed by manually counting immunolabeled neurons. The evaluation of immunohistochemical detection of β-amyloid precursor protein (βAPP) was performed by manually counting immunolabeled axons. The evaluation of immunohistochemical detection of glial fibrillary acidic protein (GFAP) in the hippocampus was performed manually by counting immunolabeled cells. For evaluation of Iba-1 and NeuN immunolabeled brain- and spinal cord sections, tissue slides were digitalized using an Olympus SLIDEVIEW VS200 scanner unit. The percentage of positive area was calculated for Iba1 and spinal cords immunolabeled for NeuN within the regions of interest (**Figure 1**) by setting an optical threshold using the QuPath-software (70–72). For hippocampi immunolabeled for NeuN, the number of positive cells in the CA 2 region of the hippocampus was calculated per 10^4^ μm^2^ using the QuPath-software and compared by neuronal density.

### Statistics, graphs and figures

Statistical analysis was performed using SAS/STAT software, Version 9.1 of the SAS^®^ System for Windows (SAS Institute Inc.) and SPSS for Windows, version 27 (IBM^®^ SPSS Chicago, IL, United States).

Significance values at 7- and 14 dpi were compared *via* multiple Wilcoxon Tests with Dwass, Steel, Critchlow-Fligner method as a *post-hoc* test to correct for multiple comparisons. Statistical significances between euthanized OT-I and OT-II mice were calculated *via* Mann-Whitney U test. Correlations were calculated *via* Spearman test. Graphs were plotted using GraphPad Prism 9 and combined into figures *via* GIMP 2.10.32.

## Results

### Clinical and neurological investigation

**Figure 2:**
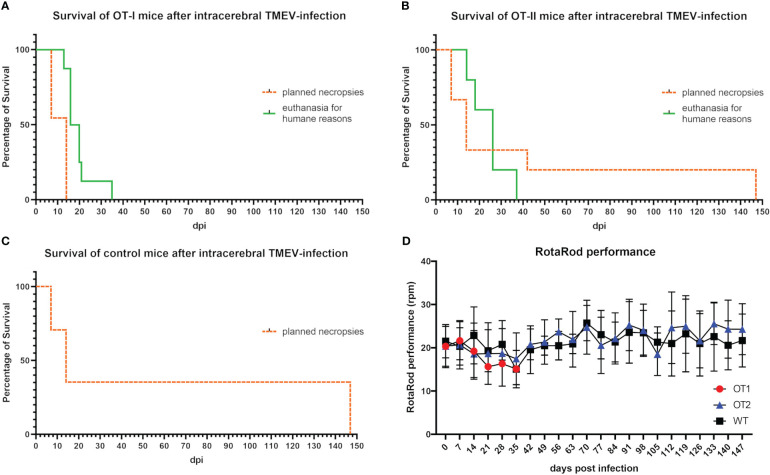
Survival curve and RotaRod performance of mice 0 to 147 days post TMEV-infection (dpi). **(A)** 12 of 19 OT-I mice reached the planned necropsy dates (dotted orange line) at 7 dpi (n=6) and 14 dpi (n=6). All OT-I mice planned for an investigation period longer than 14 dpi (n=8) had to be euthanized earlier for humane reasons (green line) between 13 and 35 dpi (13 dpi n=1, 16 dpi n=3, 20 dpi n=2, 21 dpi n=1, 35 dpi n=1). **(B)** 15 of 20 OT-II mice reached the planned necropsy dates (dotted orange line) at 7 dpi (n=5), 14 dpi (n=5), 42 dpi (n=2) and 147 dpi (n=3). Five OT-II mice had to be euthanized for humane reasons between 14 and 37 dpi (14 dpi n=1, 18 dpi n=1, 26 dpi n=2, 37 dpi n=1) (green line). **(C)** All 17 control mice with a C57BL/6 wild type phenotype (WT) reached the planned necropsy dates (dotted orange line) at 7 dpi (n=5), 14 dpi (n=6) and 147 dpi (n=6). Kaplan Meier plot. **(D)** RotaRod performance of OT mice did not decrease compared to control mice. Both OT-I mice (red dots) and OT-II mice (blue triangles) display no significant difference in the progression of RotaRod performance compared to control mice (black squares). Progression curve with standard deviation.

OT-I mice started to display signs of early neurological dysfunction characterized by mild ataxia and gait insecurities (**Table 1**) between 7 and 14 dpi. The clinical signs of these animals progressed over the course of 5 to 8 days and animals showed weight loss equal to or exceeding 20%, as well as final paralysis of one or two hind limbs, leading to euthanasia for humane reasons prior to the planned necropsies (42 and 147 dpi). In total eight OT-I mice were euthanized at 13 dpi (n=1♂, hind limb paresis), 16 dpi (n=1♂, 2♀, hind limb paresis), 20 dpi (n=2♂, hind limb paresis), 21 dpi (n=1♂, hind limb paresis) and 35 dpi (n=1♀, weight loss), respectively (**Figure 2A**).

Twelve out of 20 OT-II mice (60%) developed clinical disease characterized by mild ataxia and gait insecurities first appearing between 7 and 14 dpi. Three out of twelve OT-II mice with clinical disease (25%) made a complete clinical recovery after mild initial signs of gait insecurities and ataxia. Five OT-II mice (25% of all OT-II mice) displayed severe clinical disease, characterized by hind limb paralysis and weight loss equal to or exceeding 20% and were euthanized for humane reasons at 14 dpi (n=1♀, weight loss), 18 dpi (n=1♀, hind limb paresis), 26 dpi (n=1♂ (weight loss, 1♀ hind limb paresis) and 37 dpi (n=1♂, weight loss) respectively (**Figure 2B**). The remaining 4 OT-II mice with mild clinical disease were part of the 14 dpi study group and were sacrificed and necropsied as planned at 14 dpi.

TMEV-infected control mice (n=17) did not display any clinical disease until 7, 14 or 147 dpi, respectively (**Figure 2C**).

There was no significant difference in RotaRod performance between OT-I, OT-II and WT mice up to 35 dpi (**Figure 2D**). Mice with hind limb paresis/paralysis were not subjected to the RotaRod test on the day of necropsy. Motor function in unaffected or recovered OT-II mice (n= 11) as well as WT controls, assessed by RotaRod performance test, did not decrease significantly until 14 dpi or 147 dpi, respectively (**Figure 2D**).

All eight OT-I mice (5♂, 3♀) planned for study groups after 14 dpi were euthanized for humane reasons with no significant difference in survival times between males and females. Out of 10 OT-II mice planned for 42 and 147 dpi respectively, five (2♂, 3♀) were euthanized for humane reasons, while the other five survived to 42 and 147 dpi respectively (1♂, 4♀), without any sex-related differences.

### Histological examination

**Figure 3:**
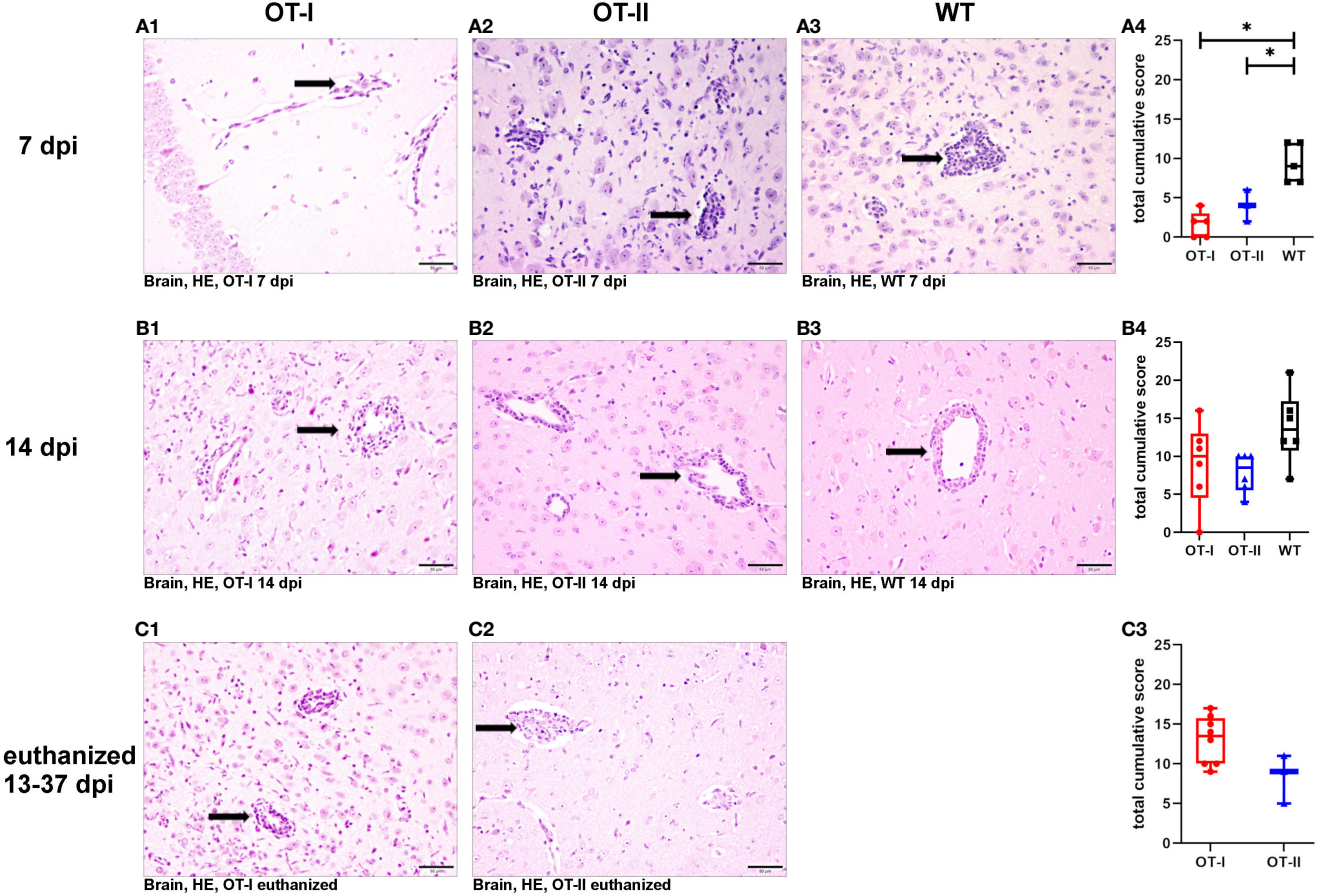
Perivascular lymphohistiocytic infiltrates in the brain. Scoring of perivascular infiltration of lymphocytes and macrophages/microglia (arrows) in HE-stained tissue sections of the brain of Theiler’s murine encephalomyelitis virus (TMEV) infected OT-I and OT-II mice as well as C57BL/6 wild type (WT) control mice. **(A1-A4)** At 7 dpi, both OT-I and OT-II mice displayed significantly reduced perivascular lymphohistiocytic infiltrates compared to control mice (OT-T vs. WT: p= 0.022, OT-II vs. WT: p= 0.22). **(B1-B4)** By 14 dpi the perivascular lymphohistiocytic infiltrates in OT-I **(B1, B4)** and OT-II mice **(B2, B4)** were increased to similar levels compared to control mice **(B3, B4)**. **(C1-C3)** In animals euthanized for humane reasons at 13-37 dpi, there was no significant difference in perivascular lymphohistiocytic infiltrates between OT-I and OT-II mice. Data are presented in box and whiskers plots (min-max) with mean and all data points. Perivascular lymphohistiocytic infiltrates (→). Bars (A2-C4) = 50 μm. Hematoxylin and eosin staining (HE). *marks statistically significant differences with p<0.05.

At 7 dpi, OT-I and OT-II mice displayed statistically significant decreased levels of perivascular lymphohistiocytic infiltrates compared to WT mice using HE stained sections (**Figures 3A1, A2, A4**). By 14 dpi, the perivascular immune cell infiltration increased drastically and reached similar levels in OT-I, OT-II and WT mice (**Figures 3B1–B4**). OT-I mice at the end stage of the disease, characterized by weight loss of 20% or more and hind limb paralysis, showed perivascular lymphohistiocytic infiltrates at a similar level to OT-II mice at the same stage of clinical disease (**Figures 3C1–C3**).

Looking at the hippocampus only, there was no significant difference in lymphohistiocytic perivascular infiltrates between the study groups at any time point (**Supplementary Figure 1**). In the spinal cord, OT-II mice displayed an increase in lymphohistiocytic perivascular infiltrates compared to WT mice at 14 dpi (p=0.028, **Supplementary Figure 2B**).

### Immunohistochemical examination

**Figure 4:**
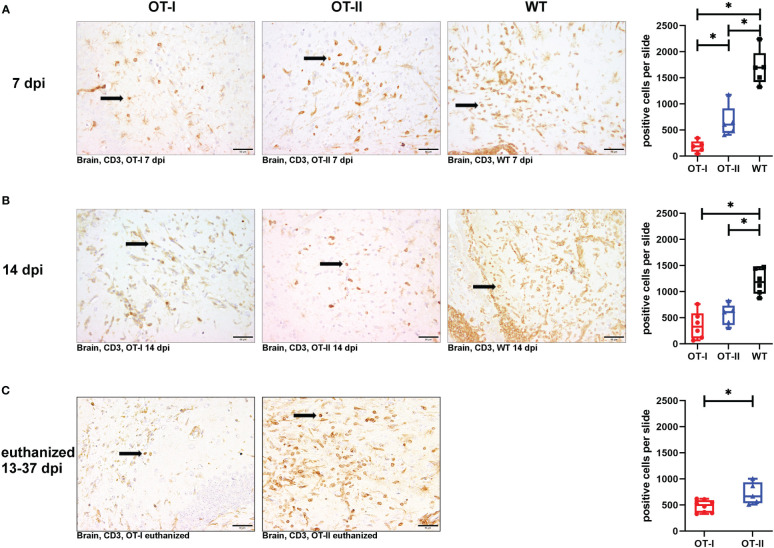
Cerebral infiltration of CD3^+^ T cells. Total numbers of CD3^+^ T cells (arrows) in the brain of Theiler’s murine encephalomyelitis virus (TMEV) infected OT-I and OT-II mice as well as C57BL/6 wild type (WT) control mice. **(A1-A4)** OT mice display decreased and delayed cerebral T cell infiltration compared to WT mice. At 7 dpi, there is significantly less T cell infiltration in OT-I and OT-II mice compared to wild type controls (WT) (OT-I vs. WT: p = 0.025, OT-II vs. WT: p = 0.025). Further, OT-I show significantly reduced T cell infiltration compared to OT-II (p = 0.025) at 7 dpi. **(B1-B4)** At 14 dpi, both OT-mouse strains display significantly reduced levels of cerebral T cell infiltration compared to WT (OT-I vs. WT: p = 0.011, OT-II vs. WT: p = 0.017). **(C1-C3)** In animals euthanized for humane reasons at 13-37 dpi, OT-II mice display a significantly increased T cell infiltration compared to OT-I mice (p = 0.045) **(C1-C3)**. Data are presented in box and whiskers plots (min-max) with mean and all data points. T cells (→). Bars **(A2-C3)** = 50 μm. ABC-DAB-immunohistochemistry, CD3, polyclonal. *marks statistically significant differences with p<0.05.

At 7 dpi, OT-I mice displayed a statistically significant reduction in CD3^+^ T cell infiltration into the brain compared to both OT-II and control animals (**Figures 4A1–A4**). By 14 dpi, OT-I mice displayed a significant reduction in the infiltration of CD3^+^ T cells only compared to control mice (**Figures 4B1–B4**). Upon reaching the defined criteria for euthanasia, OT-II mice displayed stronger CD3^+^ T cell infiltration, compared to OT-I mice at a similar stage of disease (**Figures 4C1–C3**).

OT-II mice at 7 dpi showed significantly increased T cell infiltration into the brain than OT-I mice, but significantly less infiltration than control mice (**Figures 4A1–A4**). At 14 dpi, the infiltration of CD3^+^ T cells in OT-I and OT-II mice is no longer statistically significant different, but it was still on a significantly lower level compared to control mice (**Figures 4B1–B4**). Upon reaching the defined criteria for euthanasia, OT-II mice displayed increased CD3^+^ T cell infiltration, compared to OT-I mice at a similar stage of disease (**Figures 4C1–C3**).

Looking at the hippocampus only, OT-II mice displayed an increased CD3^+^ T cell infiltration at 7 dpi compared to OT-I mice (p=0.043, **Supplementary Figure 3A**). By 14 dpi, OT-I and OT-II mice displayed a similar degree of CD3^+^ T cell infiltration with WT mice displaying significantly increased infiltration compared to OT-II mice (p=0.046, **Supplementary Figure 3B**). In the spinal cord, WT mice displayed an increased CD3 reaction at 7 dpi compared OT-I mice (p=0.025, **Supplementary Figure 4A**). In animals euthanized for humane reasons at 13-37 dpi, OT-II mice displayed an increased CD3^+^ T cell infiltration compared to OT-I mice (p=0.019, **Supplementary Figure 4C**).

**Figure 5:**
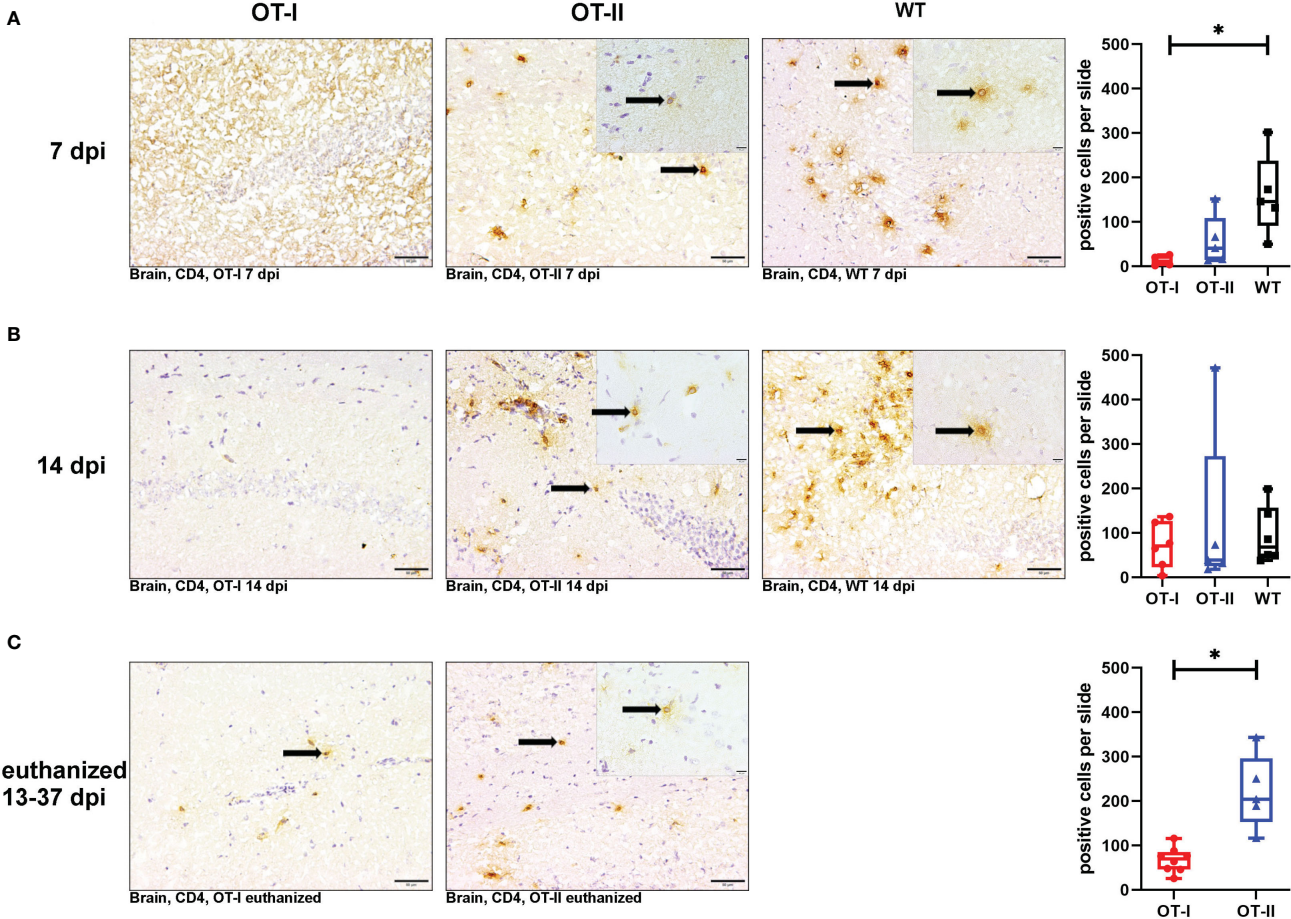
Cerebral infiltration of CD4^+^ T cells. Total numbers of CD4^+^ T cells (arrows) in the brain of Theiler’s murine encephalomyelitis virus (TMEV) infected OT-I and OT-II mice as well as C57BL/6 wild type (WT) control mice. Inserts display higher magnifications. **(A1-A4)** At 7 dpi, OT-I mice displayed significantly reduced CD4^+^ T cell infiltration into the brain compared to control mice (WT) (p = 0.038). OT-II mice displayed no significant difference in CD4^+^ T cell infiltration compared to either OT-I or WT mice. **(B1-B4)** At 14 dpi, all groups showed similar levels of cerebral infiltration of CD4^+^ T cells. **(C1-C3)** In euthanized animals, OT-II mice displayed increased CD4^+^ T cell infiltration compared to OT-I mice at the same stage of disease (p = 0.002). Data are presented in box and whiskers plots (min-max) with mean and all data points. CD4^+^ T cells (→). Bars **(A1-C2)** = 20 μm. Bars in inserts = 10 μm. ABC-DAB-immunohistochemistry, CD4, monoclonal. *marks statistically significant differences with p<0.05.

At 7 dpi, the number of CD4^+^ T cells in the brains of OT-I mice was significantly reduced compared to control mice, whereas the number of CD4^+^ T cells in OT-II mice reached an intermediate level between the one observed in OT-I mice and control mice without statistically significant difference to either of them (**Figures 5A1–A4**). At 14 dpi, the cerebral infiltration of CD4^+^ T cells had reached similar levels across all study groups (**Figures 5B1–B4**). At the end stage of disease, OT-II mice displayed a significant increase in CD4^+^ T cell infiltration compared to OT-I mice at the same stage of disease (**Figures 5C1–C3**).

Looking at the hippocampus only, WT mice displayed an increased CD4^+^ T cell reaction compared to OT-I mice at 7 dpi (p=0.036, **Supplementary Figure 5A**). At other time points, there were no differences between the study groups (**Supplementary Figure 5**). In the spinal cord, there was no significant difference in CD4^+^ T cell infiltration between the study groups at any time point (**Supplementary Figure 6**).

**Figure 6:**
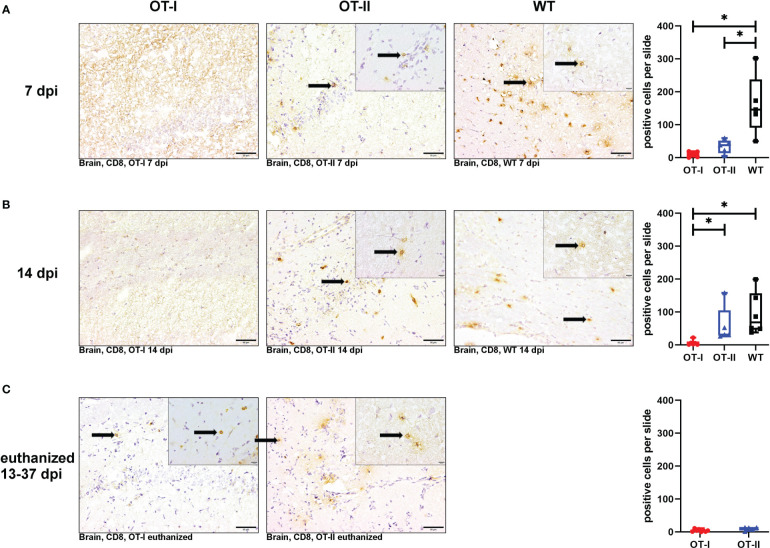
Cerebral infiltration of CD8^+^ T cells. Total numbers of CD8^+^ T cells (arrows) in the brain of Theiler’s murine encephalomyelitis virus (TMEV) infected OT-I and OT-II mice as well as C57BL/6 wild type (WT) control mice. Inserts display higher magnifications. **(A1-A4)** Both OT-I and OT-II mice at 7 dpi displayed a significant reduction in cerebral CD8^+^ T cell infiltration compared to WT mice (OT-I vs. WT: p = 0.025, OT-II vs. WT: p = 0.043). **(B1-B4)** At 14 dpi, OT-I mice displayed lower levels of CD8^+^ T cell infiltration compared to both OT-II and WT mice (OT-I vs. OT-II: p= 0.028, OT-I vs. WT: p = 0.01). **(C1-C3)** In animals euthanized for humane reasons at 13-37 dpi, there is no significant difference between OT-I and OT-II mice in cerebral infiltration with CD8^+^ T cells **(C1-C3)**. Data are presented in box and whiskers plots (min-max) with mean and all data points. CD8^+^ T cells (→). Bars **(A1-C2)** = 20 μm. Bars in inserts = 10 μm. ABC-DAB-immunohistochemistry, CD8, monoclonal. *marks statistically significant differences with p<0.05.

At 7 dpi, the number of CD8^+^ T cells in the brains of OT-I and OT-II mice was significantly reduced compared to control mice (**Figures 6A1–A4**). At 14 dpi, OT-I mice displayed a reduced infiltration of CD8^+^ T cells compared to both OT-II and control mice without significant difference between OT-II and control mice (**Figures 6B1–B4**). At the end stage of the clinical disease, OT-I as well as OT-II mice showed very low numbers of CD8^+^ T cell infiltration into the brain, with no statistically significant difference between these groups (**Figures 6C1–C3**).

Looking at the hippocampus only, both OT-II and WT mice displayed an increased CD8^+^ T cell infiltration compared to OT-I mice at 7 dpi (OT-I vs. OT-II p=0.021, OT-I vs. WT p=0.022, **Supplementary Figure 7A**). WT mice still displayed an increased CD8^+^ T cell infiltration at 14 dpi compared to OT-I mice (p=0.01, **Supplementary Figure 7B**). In the spinal cord, there was no significant difference in CD8^+^ T cell infiltration between the study groups at any time point (**Supplementary Figure 8**).

**Figure 7:**
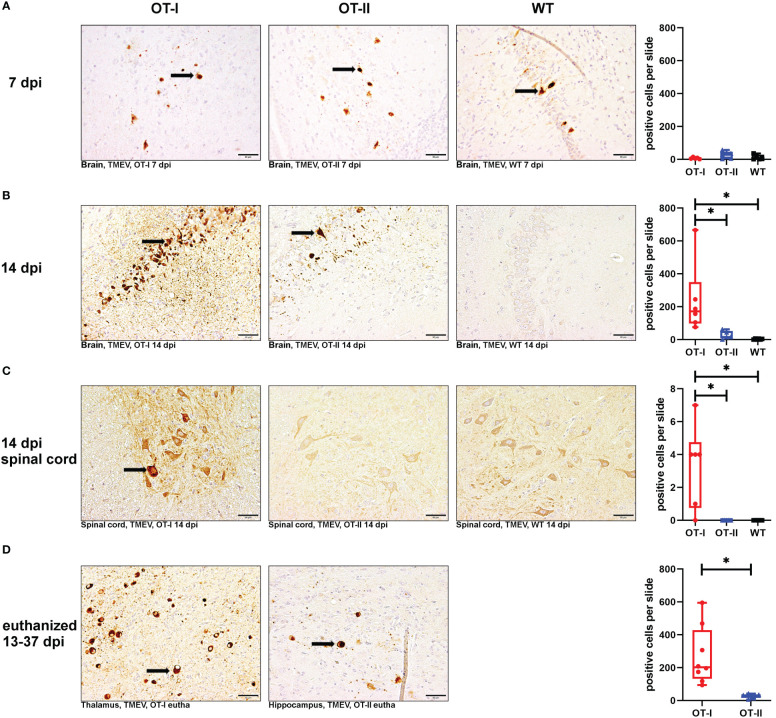
Theiler’s murine encephalomyelitis virus (TMEV)-immunoreactivity in the brain and spinal cord. Total numbers of TMEV antigen-positive cells (arrows) in the brain and spinal cord of TMEV-infected OT-I and OT-II mice as well as C57BL/6 wild type (WT) control mice. **(A1-A4)** At 7 dpi, all groups display similar levels of TMEV-positive cells in the brain. **(B1-B4)** OT-I mice display an increased cerebral virus load at 14 dpi compared to WT- and OT-II mice (OT-I vs. OT-II: p = 0.017, OT-I vs. WT: p =0.01). **(C1-C4)** Only OT-I mice showed virus spread to the spinal cord starting at 14 dpi, leading to significantly increased spinal virus immunoreactivity compared to both OT-II and WT mice (OT-I vs. OT-II: p = 0.046, OT-I vs. WT: p = 0.046). **(D1-D3)** In animals euthanized for humane reasons at 13-37 dpi, OT-I mice still showed increased virus load compared to OT-II mice (p = 0.002). Data are presented in box and whiskers plots (min-max) with mean and all data points. TMEV-positive cells (). Bars **(A1-D2)** = 50 μm. ABC- DAB- immunohistochemistry, TMEV, polyclonal. *marks statistically significant differences with p<0.05.

To assess the viral clearance by the immune system, we investigated the cerebral virus antigen expression at the different time points. There was no statistically significant difference between study groups regarding cerebral virus immunoreactivity at 7 dpi (**Figures 7A1–A4**). At 14 dpi, control mice had almost completely cleared TMEV from the CNS while OT-I mice still displayed a high virus antigen load within the brain. The strongest immunoreactivity was detected in hippocampus (**Supplementary Figure 10**) and thalamus (data not shown). Although OT-II mice had not completely cleared the virus from the brain by 14 dpi, the cerebral virus immunoreactivity was significantly lower than in OT-I mice and not statistically different from WT mice (**Figures 7B1–B4**). Furthermore, in contrast to OT-II and WT mice, OT-I mice displayed virus spread to the spinal cord by 14 dpi (**Figures 7C1–C4**; **Supplementary Figure 9**). In animals euthanized for humane reasons, OT-I mice still showed significantly higher cerebral virus loads than OT-II mice with the latter having almost cleared the virus (**Figures 7D1–D3**).

Looking at the hippocampus only, OT-I mice displayed an increased virus immunoreactivity at 14 dpi compared to WT mice (p=0.015, **Supplementary Figure 10B**). In animals euthanized for humane reasons at 13-37 dpi, OT-I mice displayed an increased virus immunoreactivity compared to OT-II mice (p=0.018, **Supplementary Figure 10C**).

**Figure 8:**
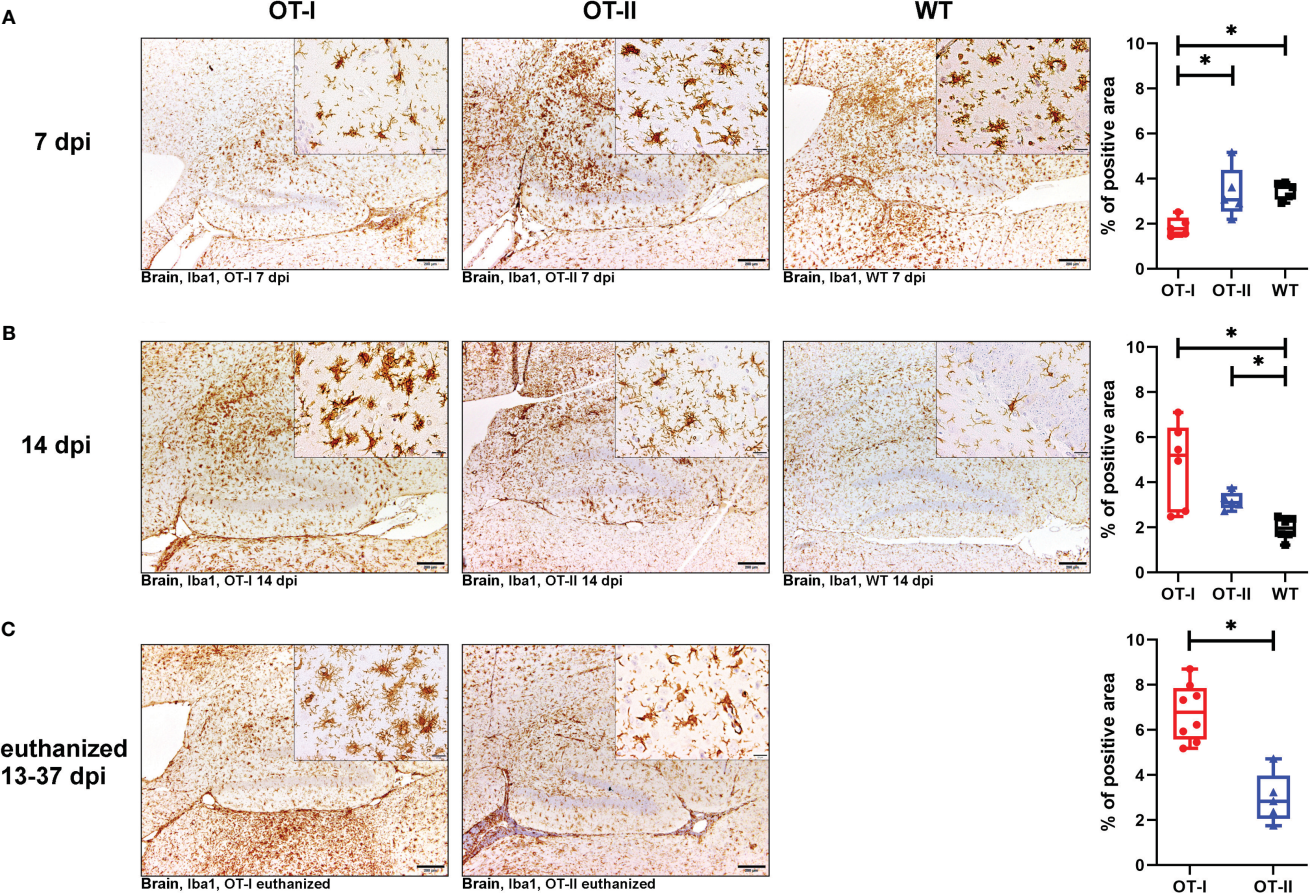
Microgliosis and macrophage infiltration in the brain measured by detection of Iba1. Percent (%) of area occupied by Iba1 labelled cells in the brain of Theiler’s murine encephalomyelitis virus (TMEV) infected OT-I and OT-II mice as well as C57BL/6 wild type (WT) control mice. **(A1-A4)** At 7dpi, OT-I mice display lower levels of microglia/macrophages proliferation compared to WT approaching statistical significance (p = 0.065). OT-II mice display relatively constant levels of microglia/macrophage activation and proliferation from 7 dpi to euthanasia for humane reasons. At 7 dpi, microglia/macrophage numbers in OT-II mice are similar to WT- and OT-I mice. OT-I mice at 7 dpi mainly displayed mainly resting microglia, while OT-II mice showed a mixture of resting- and amoeboid microglia. Control mice at 7 dpi mainly showed amoeboid microglia. **(B1-B4)** At 14 dpi, microgliosis in OT-I mice is not significantly different from OT-II mice and significantly stronger than in WT mice (p = 0.029). Microglial activity in WT mice at 14 dpi is significantly lower than in to OT-II mice at 14 dpi (p = 0.038). Microglia morphology in OT-I mice changed to them mainly displaying the amoeboid phenotype. OT-II mice at 14 dpi displayed a mixture of resting- and amoeboid microglia. Control mice showed a change in morphology towards resting microglia by 14 dpi. **(C1-C3)** Microgliosis in OT-I mice euthanized for humane reasons is significantly stronger compared to OT-II mice at the same stage of disease (p = 0.002). In animals euthanized for humane reasons, OT-I mice displayed a mixture of amoeboid- and hyper-ramified microglia, while OT-II mice displayed a mixture of resting- and amoeboid microglia **(C2, C3)**. Data are presented in box and whiskers plots (min-max) with mean and all data points. Bars **(A1-C2)** = 200 μm. Bars in inserts = 20 μm. ABC- DAB- immunohistochemistry, ionized calcium-binding adapter molecule 1 (Iba1), polyclonal. *marks statistically significant differences with p<0.05.

At 7 dpi, OT-I mice displayed a diminished cerebral microglia/macrophage activation, that lacked statistical significance compared to WT mice (**Figures 8A1–A4**). By 14 dpi, this trend was reversed; at this time point OT-I and OT-II mice displayed higher numbers of intracerebral microglia/macrophages than WT mice (**Figures 8B1–B4**). Microglia/macrophage counts in OT-I mice further increased in animals euthanized for humane reasons, displaying significantly higher numbers of microglia/macrophages compared to OT-II mice at the same stage of disease (**Figures 8C1–C3**).

OT-II mice at 7 dpi displayed microglia/macrophage-levels similar to WT (**Figure 8A1–A4**), and this level stayed constant between 7 and 14 dpi. Accordingly, at 14 dpi they displayed microglia/macrophage numbers similar to OT-I and significantly higher than WT mice, for which microglia/macrophage numbers declined between 7 and 14 dpi (**Figures 8B1–B4**). Microglia/macrophage numbers in OT-II mice also remained steady between 14 dpi and later time points on which the animals were euthanized for humane reasons. Accordingly, microgliosis in OT-II mice euthanized for humane reasons was significantly lower than in OT-I mice at the same disease stage (**Figures 8C1–C3**).

Iba-1 positive microglia in OT-I mice remained phenotypically unchanged compared to controls until 7 dpi (**Figure 8A2**). Afterwards they exhibited amoeboid and hyper-ramified phenotype (**Figures 8B2, C2**). Microglia morphology in OT-II mice did not change as drastically over the course of the experiment. At 7 dpi, OT-II mice mainly showed resting-, ramified- and amoeboid microglia in equal measure (**Figure 8A3**). This morphology remained to be constant throughout the course of the disease (**Figures 8B3, C3**). WT mice displayed mainly amoeboid microglia until 7 dpi (**Figures 8A4**). By 14 dpi, parallel to a reduction in overall numbers of microglia/macrophages, their morphology changed to mainly resting microglia (**Figure 8B4**).

Looking at the hippocampus only, OT-I mice displayed an increased microgliosis and macrophage infiltration compared to WT mice at 14 dpi (p=0.043, **Supplementary Figure 11B**). In the spinal cord, OT-I mice displayed an increased microgliosis and macrophage infiltration compared to both OT-II and WT mice at 14 dpi (OT-I vs. OT-II p=0.017, OT-I vs. WT p=0.011, **Supplementary Figure 12B**). In animals euthanized for humane reasons at 13-37 dpi, OT-I mice also displayed an increased spinal cord microgliosis and macrophage infiltration compared to OT-II mice (p=0.015, **Supplementary Figure 12C**).

**Figure 9:**
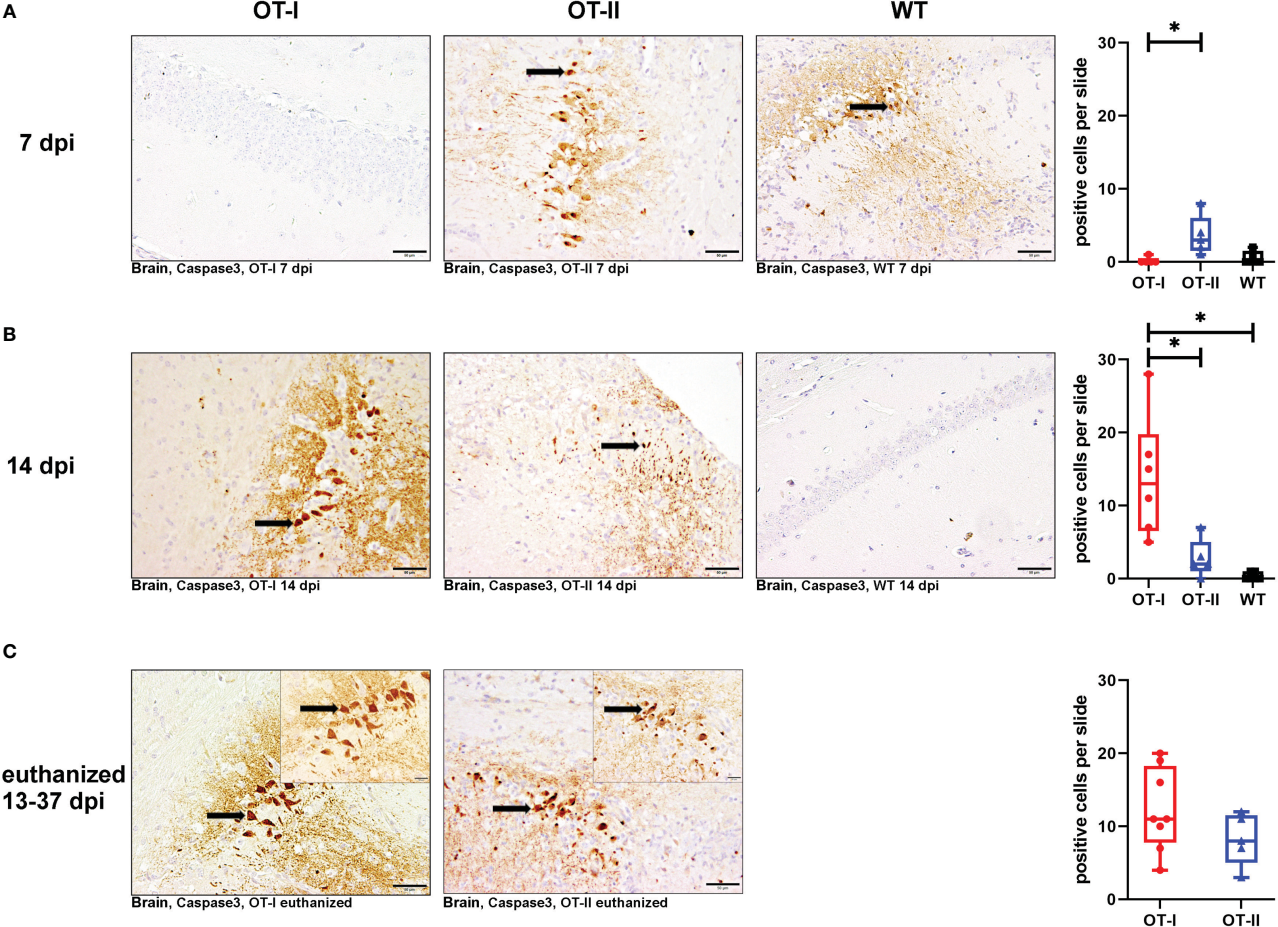
Neuronal apoptosis in brain measured by positive cleaved caspase 3 signalling. Total numbers of cleaved caspase 3- positive, apoptotic cells (arrows) in the brain of Theiler’s murine encephalomyelitis virus (TMEV) infected OT-I and OT-II mice as well as C57BL/6 wild type (WT) control mice. **(A1-A4)** At 7 dpi, OT-II mice show an increase in neuronal apoptosis compared to OT-I (p = 0.026), but not WT. **(B1-B4)** At 14 dpi, OT-I mice show an increase in neuronal apoptosis compared to both WT and OT-II (OT-I vs. OT-II: p = 0.035, OT-I vs. WT: p = 0.009). **(C1-C3)** All mice euthanized for humane reasons at 13-37 dpi displayed similar levels of neuronal apoptosis. Data are presented in box and whiskers plots (min-max) with mean and all data points. Cleaved caspase 3-positive neurons (). Bars **(A1-C2)** = 50 μm. Bars in inserts **(C1, C2)** = 20 μm. ABC- DAB- immunohistochemistry, cleaved caspase 3, polyclonal. *marks statistically significant differences with p<0.05.

OT-I mice at 7 dpi displayed significantly less neuronal apoptosis in the brain compared to OT-II mice, but no difference compared to WT mice (**Figures 9A1–A4**). At 14 dpi this was switched around, as by then OT-I mice displayed a statistically significant increase in neuronal apoptosis, compared to both OT-II and control mice (**Figures 9B1–B4**). Upon euthanasia for humane reasons, OT-I and OT-II mice displayed similar levels of cerebral neuronal apoptosis (**Figures 9C1–C3**). Hippocampus (**Supplementary Figure 13**) and thalamus (data not shown) displayed the highest numbers of cleaved caspase3-positive neurons. The neuronal apoptosis in OT-II mice at 7 dpi was significantly increased, compared to OT-I mice, but not higher than in WT mice (**Figures 9A1–A4**). By 14 dpi, OT-II mice showed a lower level of neuronal apoptosis compared to OT-I mice, but not significantly different from WT mice (**Figures 9B1–B4**).

Neuronal apoptosis in OT-I mice correlated moderately with the amount of detectable TMEV-antigen (coefficient: 0.77, p=0.0001) and, to a lesser degree, with microgliosis (coefficient: 0.586, p=0.008). In OT-II mice, neuronal apoptosis correlated more strongly with infiltration of CD4^+^ T cells (coefficient: 0.741, p=0.00018) and not as strongly with detection of TMEV-antigen (coefficient: 0.6887, p=0.0008).

Looking at the hippocampus only, all study groups displayed a similar degree of neuronal apoptosis at all time points (**Supplementary Figure 13**). No mouse displayed neuronal apoptosis in the spinal cord at any time point (**Supplementary Figure 14**).

All study groups displayed a similar degree of glial fibrillary acidic protein (GFAP) positive cells in the hippocampus at all time points (**Supplementary Figure 15**).

**Figure 10:**
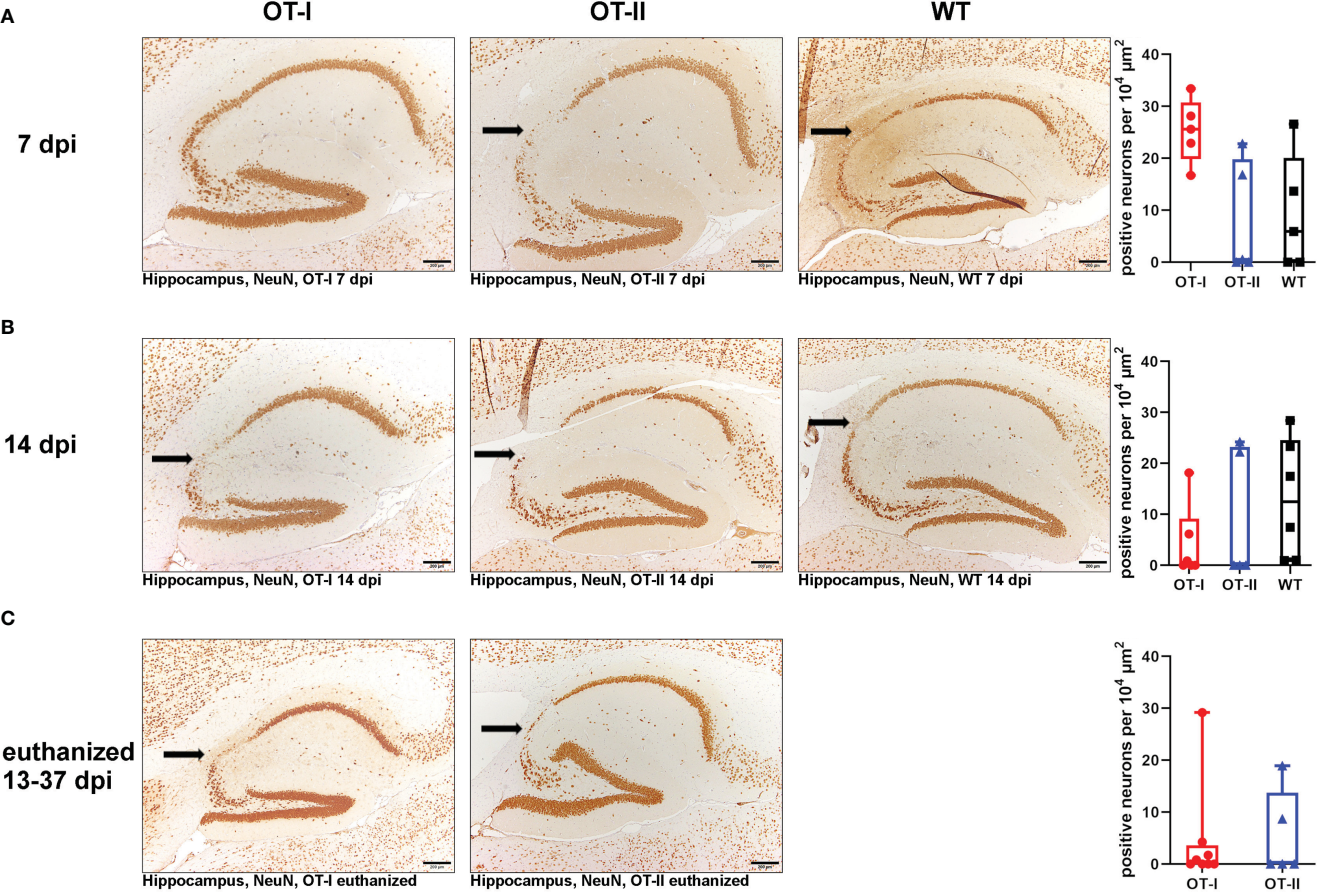
Hippocampal NeuN-positive neuronal density in the CA2 region. Relative numbers of NeuN-positive neurons per area (10^4^ µm^2^) in the hippocampal CA2 region (arrow) of Theiler’s murine encephalomyelitis virus (TMEV) infected OT-I and OT-II mice as well as C57BL/6 wild type (WT) control mice. **(A1-A4)** At 7 dpi, all groups showed a similar NeuN-positive neuronal density in the CA2 region of the hippocampus without significant differences. **(B1-B4)** At 14 dpi, all groups showed a similar NeuN-positive neuronal density in the CA2 region of the hippocampus without significant differences. **(C1-C3)** In animals euthanized for humane reasons at 13-37 dpi, OT-I and OT-II mice displayed similar loss of NeuN-positive neurons without significant differences. Data are presented in box and whiskers plots (min-max) with mean and all data points. Loss of NeuN-positive cells in CA2 region (). Bars **(A1-C2)** = 200 μm. ABC-DAB-immunohistochemistry, neuronal nuclear protein (NeuN), monoclonal.

Hippocampal neuronal density in the CA2 region, as measured by expression of NeuN remained similar between all study groups at all time points (**Figure 10**). Neuronal loss mainly affected the hippocampal CA2 region (**Figure 10**). In the spinal cord, there were no differences in neuronal NeuN expression between the study groups at any time point (**Supplementary Figure 16**).

Neither OT-I, OT-II nor control mice displayed axonal damage within the brain, hippocampus or spinal cord at any time point (**Supplementary Figures 17–19**).

## Discussion

The aim of this study was the evaluation and comparison of disease progression and clinical outcome, virus persistence, alterations in T cell response, microglial activation, neuronal apoptosis, and occurrence of demyelination as well as axonal damage in OT-I and OT-II mice after intracerebral infection with TMEV-BeAn, compared to control C57BL/6 mice.

All control mice were able to clear the virus and reached the planned end-points of necropsy without developing severe disease symptoms, whereas all OT-I mice and 25% of OT-II mice had to be euthanized for humane reasons within 37 dpi. Animals were euthanized due to either hind limb paresis (OT-I: n=7, OT-II: n=2) or weight loss equal to or exceeding 20% (OT-I: n=1, OT-II: n=3). Clinical disease in OT-I and OT-II mice was not associated with motor dysfunction before the onset of hind limb paresis. OT-I mice displayed large quantities of TMEV-antigen in the brain starting at 14 dpi compared to control mice, indicating that virus clearance was severely impaired in the subacute phase after infection. Similarly, a lethal outcome has been described for OT-I mice after infection with TMEV-DA, where the mice died between 12 and 17 dpi (57). A similar clinical outcome can also be seen after infection of susceptible mouse strains with TMEV-GDVII (27). There, a lethal acute disease is caused by extensive neuronal damage by this exclusively neurotropic TMEV-strain, with a concurrent reduction in the antiviral immune response (27). Conversely, in susceptible mouse strains, TO viruses induce a mainly T cell mediated immune response and a change in tropism over the course of the disease from neurons to astrocytes and oligodendrocytes with low virus levels being observed in the chronic phase of the infection (20). The severe clinical disease observed in the present study was associated with high levels of intracerebral TMEV-antigen and an elevation of neuronal cell death within the brain of OT-I mice at 14 dpi and at later time points, with virus even spreading into the spinal cord in OT-I mice. This indicates that these mice were unable to clear the virus, not even with delayed kinetics. Moreover, neuronal apoptosis was correlated with the strongest intracerebral virus immunoreactivity. We therefore suspect that, similar to GDVII infection, disease in TMEV-BeAn infected OT-I mice is mainly caused by extensive neuronal damage due to a direct viral cytolytic effect (27). The increased survival time in the present study, compared to TMEV-DA infected OT-I mice, may be due to the BeAn strain causing, in general, less neuronal damage in the early stage of disease, compared to TMEV-DA (73).

The T cell infiltration in the brain was significantly less pronounced in both OT-I and OT-II mice compared to WT mice. An effective immune response to TMEV-TO infection depends on the ability to mount a cytotoxic CD8^+^ T cell response (35, 43–48). In OT-I mice, the OVA specificity of the majority of CD8^+^ T cells precludes mounting a TMEV-antigen specific CD8^+^ immune response (54). Therefore, the infiltration of cytotoxic CD8^+^ T cells stays low during the whole observation period accompanied by the unability to effectively clear the virus. In GVII-infection, clinical disease in susceptible mice is also associated with a severely reduced immune response (26, 27). OT-II mice harbour a numerically reduced CD8^+^ T cell compartment (53), and part of their CD8^+^ T cells might also express an OVA-specific TCR. Nevertheless, CD8^+^ T cells infiltrate the brain with slightly delayed kinetics compared to WT mice (day 14) and are able to clear the virus at this time point. OT-II mice with a negative clinical outcome still had hardly any detectable virus immunoreactivity. Therefore, it seems unlikely that their condition was due to direct cytolytic virus effects. Surprisingly, these OT-II mice displayed elevated numbers of CD4^+^ T helper cells in the brain. An enhanced CD4^+^ T cell reaction has already been shown to contribute to pathology in TMEV-IDD in the way of a delayed-type hypersensitivity reaction (20, 37, 38, 74). Therefore, we believe that the disease in OT-II mice with a severe course is rather due to the increase of infiltrating CD4^+^ T cells in the late phase of disease, leading to a secondary (auto-) immune response mediated by CD4^+^ helper T cells rather than their inability to clear the virus. Autoimmune phenomena are always low in penetrance (75, 76). We therefore suspect the variable clinical outcome in OT-II mice to be the result of individual variations during recombination of the TCR chains, resulting in a different TCR-repertoire in each individual mouse (53). The elevation in the number of CD4^+^ T cells may be the result of increased cytokine expression (77). At the moment, however, the factors contributing to the development of autoimmunity in individual mice remain unclear and should be investigated in future studies.

OT-I mice displayed the strongest microgliosis of any study group, starting at 14 dpi. They also displayed the highest level of neuronal apoptosis, also with a later onset than in OT-II mice. Cerebral microgliosis and microglial activation in OT-II mice remained at a steady state throughout the experiment. OT-II mice also displayed relatively constant, early onset levels of neuronal apoptosis with the only increase after 14 dpi in animals with a negative clinical outcome (p=0.032 Mann-Whitney U). In the healthy brain, the microglia population consists of three main morphological types (78). Resting (or surveilling) microglia are the main morphological phenotype of microglia (78). They have a small soma and long, ramified processes (78). Satellite microglia are in direct contact with neuronal stomata and, together with juxtavascular microglia, represent the remaining microglia population under non-inflammatory conditions (78). Pathology and activation of microglia are associated with a change in microglial morphology (78). In addition, non-activated macrophages can be found perivascularly, as well as in the choroid plexus and meninges (79, 80). In the first stage of activation, microglia typically display hyperramification, which then evolves into an amoeboid morphotype due to enlargement of the soma (78). Microglia are implicated in disease progression of many neurodegenerative diseases (80). However, in these diseases, microglia dysfunction does not appear to be the sole or initial cause of disease (80). Microglia also have a diverse role in the immune reaction to CNS infection (8). After bacterial infection, microglia recruit leukocytes to the site of infection (81). However, they have also been shown to contribute to pathology through upregulation of MHC-II (8, 82). In West Nile virus (WNV) encephalitis, microglia have also been demonstrated to contribute to chronic disease (1). They contribute to loss of hippocampal presynaptic terminals in both murine and humans WNV infection (1, 83). Similar results have been seen in TMEV-IDD, where microglia are implicated in chronic demyelination and axonal damage through bystander damage (20). Additionally, microglia and perivascular macrophages have been identified as the main cerebral APC-population responsible for the induction of a CD8^+^ T cell response *via* antigen presentation through MHC-I (4). However, virus antigen presentation *via* MHC-I fails to induce an adequate CD8^+^ T cell response in OT-I mice (54). Apoptosis has been shown to involve a terminal increase in cytosolic adenosine triphosphate (ATP) (84). ATP is then released into the surrounding tissue during apoptosis in order to induce phagocytosis (85). The release of cellular ATP through neuronal apoptosis strongly induces microglia proliferation and activation (84–86). We therefore suspect that microgliosis in OT-I mice is a secondary event to neuronal apoptosis, as well as an attempt to induce a CD8^+^ T cell reaction through antigen presentation *via* MHC-I.

We further speculate that microgliosis is also a secondary event in OT-II mice following neuronal apoptosis. However, as cerebral virus immunoreactivity is virtually absent in OT-II mice after 14 dpi, we suggest that disease in severely affected mice is propagated by immunopathology in a concerted action by microglia and CD4^+^ T cells.

Unexpectedly, no study group displayed CNS demyelination at any time point in this experiment. In addition, neither OT-I nor OT-II nor WT mice displayed significant axonal damage in the spinal cord at any time point. After infection with TMEV-TO, susceptible mouse strains like SJL develop chronic progressive, demyelinating leukomyelitis, starting at around 1 month post infection (20, 29, 87, 88). C57BL/6 mice have been shown to be resistant to demyelination after TMEV-infection (20). However, MHC-I deficient mice on a C57BL/6 background display spinal cord demyelination without associated clinical symptoms, starting at 45 dpi (49, 51). OT-I mice and OT-II mice with a negative clinical outcome did not reach this time point in this experiment, precluding an analysis of this phenomenon (49, 51). Surviving OT-II mice did not develop immunopathology and therefore do not seem to differ from C57BL/6 WT mice in their ability to prevent demyelination and axonal damage.

In previous studies, sex has been shown to influence the susceptibility to TMEV-infection in C57L, SJL mice and other mouse strains, although the mechanism is not clearly understood (89). Female C57L mice are resistant to TMEV in contrast to male C57L mice (89, 90). Furthermore, in susceptible SJL mice, males display a faster clinical progression compared to females (91). Aside from genetic variations in the H-2D locus (43), this seems to be connected to sex related genetic variations in antiviral cellular and humoral immune responses in these mouse strains, which are furthermore altered by the influence of estrogen and testosterone (92, 93). However, no effect of the sex was seen in OT mice on a C57BL/6 background in the present study. Male as well as female OT-I and OT-II mice were equally affected by clinical disease.

## Conclusions

In both, OT-I and OT-II mice, clinical disease is associated with decreased CD8^+^ T cell infiltration, underlining the importance of a cytotoxic T cell response in the TMEV-model. Furthermore, neuronal damage in OT-I mice is correlated with cerebral virus load and is most likely the result of direct viral impact. Disease in OT-II mice was associated more with microgliosis and infiltration of CD4^+^ T helper cells. Therefore, it seems that autoimmune phenomena leading to immunopathology are contributing to disease development in OT-II mice with, however, a limited penetrance.

## Data availability statement

The raw data supporting the conclusions of this article will be made available by the authors, without undue reservation.

## Ethics statement

The animal study was reviewed and approved by Niedersächsisches Landesamt für Verbraucherschutz und Lebensmittelsicherheit (LAVES), Oldenburg, Germany, permission number 33.8-42502-17/2418.

## Author contributions

FL and AF: Breeding of genetically modified mice; KH, WB, FL, and AF: Study design and manuscript editing; RW and KH: Animal experiment and necropsies; RW, WB, KH, and AR: Immunohistochemistry, histological- and immunohistochemical evaluation and data analysis; KR: Statistical analysis; RW, WB, KH, FL, and AR: Writing of the manuscript. All authors reviewed and approved the final version of the manuscript. All authors contributed to the article and approved the submitted version.
