# Supplementary material for: Ovalbumin-specific CD4+ and CD8+ T cells contribute to different susceptibility for Theiler’s murine encephalomyelitis virus persistence
Source: Front Immunol. 2023 May 24;14:1194842. doi: 10.3389/fimmu.2023.1194842 (PMC10244668; doi:10.3389/fimmu.2023.1194842)
Supplement: Supplementary file 1 [file DataSheet_1.pdf]

Supplementary Material

Supplementary Fig. 1: Perivascular lymphohistiocytic infiltrates in the hippocampus

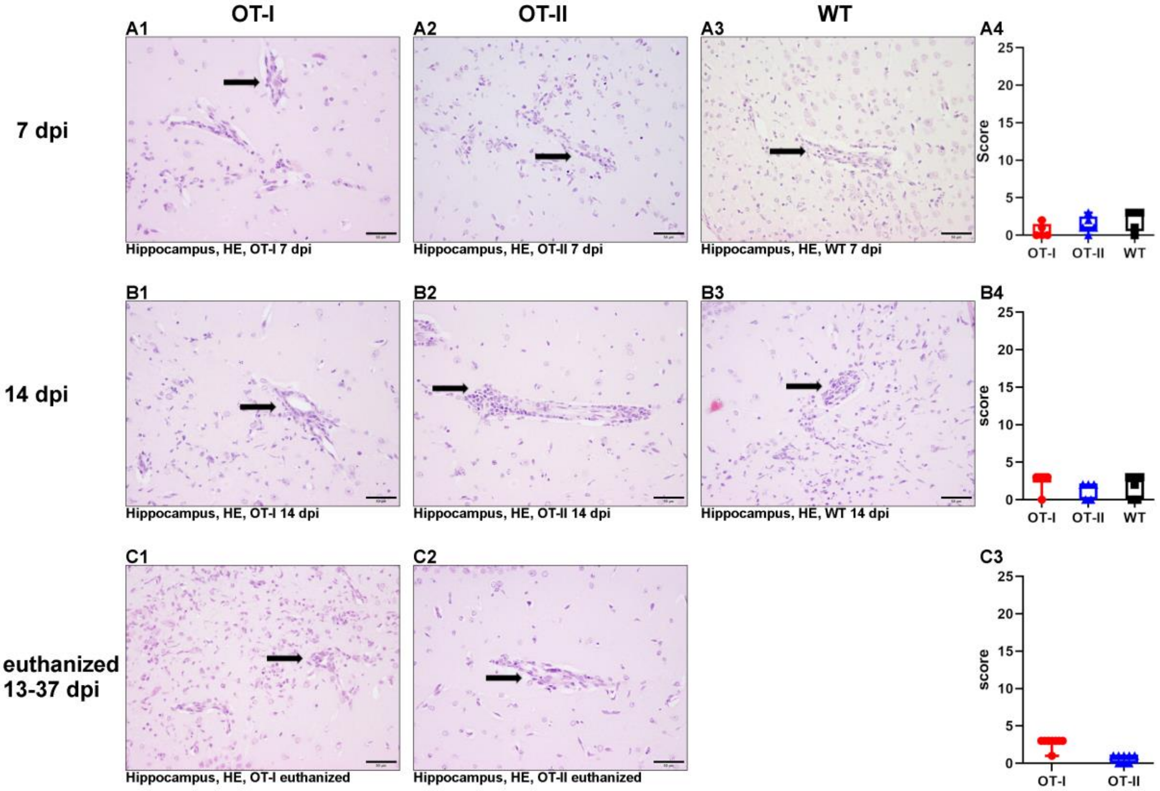

Suppl. Fig. 1: Perivascular lymphohistiocytic infiltrates in the hippocampus
Scoring of perivascular lymphohistiocytic infiltrates (arrows) in HE-stained tissue sections of the hippocampus of Theiler's murine encephalomyelitis virus (TMEV) infected OT-I and OT-II mice as well as C57BL/6 wild type (WT) control mice.

A1-A4: At 7 dpi, all groups showed a similar degree of perivascular lymphohistiocytic infiltrates without significant differences.

B1-B4: At 14 dpi, there was no significant difference between the study groups with respect to the degree of perivascular lymphohistiocytic infiltrates.

C1-C3: Animals euthanized for humane reasons at 13 - 37 dpi displayed a similar degree of perivascular lymphohistiocytic infiltrates without significant differences.

Data are presented in box and whiskers plots (min-max) with mean and all data points. Perivascular lymphohistiocytic infiltrates (➡). Bars (A1-C2) = 50 µm. Hematoxylin and eosin staining (HE).

Supplementary Fig. 2: Perivascular lymphohistiocytic infiltrates in the spinal cord

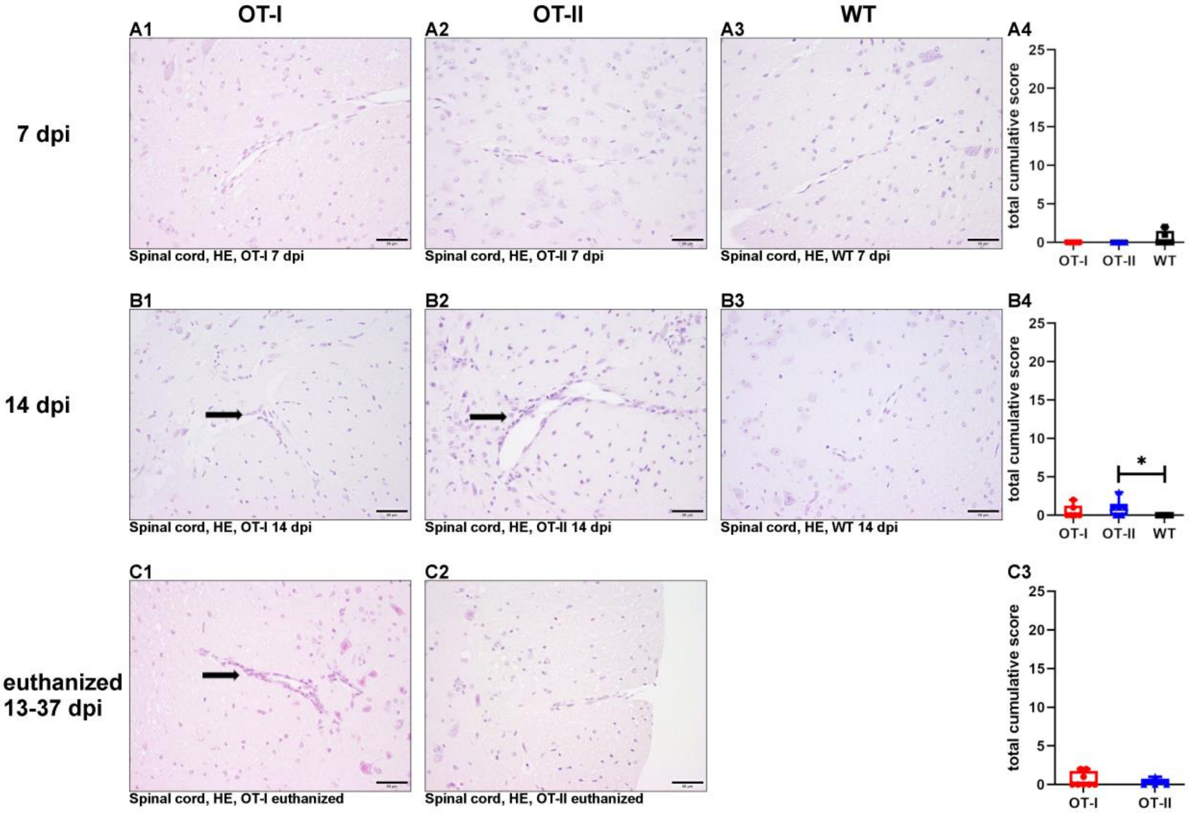

Suppl. Fig. 2: Perivascular lymphohistiocytic infiltrates in the spinal cord
Additive scoring of perivascular lymphohistiocytic infiltrates (arrows) in HE-stained tissue sections of three spinal cord areas (cervical, thoracic, and lumbar) of Theiler's murine encephalomyelitis virus (TMEV) infected OT-I and OT-II mice as well as C57BL/6 wild type (WT) control mice.

A1-A4: At 7 dpi, all groups showed a similar degree of perivascular lymphohistiocytic infiltration without significant differences.

B1-B4: At 14 dpi, OT-II mice showed a significantly higher degree of perivascular lymphohistiocytic infiltrates in the spinal cord compared to WT mice (OT-II vs. WT: p = 0.028).

C1-C3: Animals euthanized for humane reasons at 13 – 37 dpi displayed a similar degree of perivascular lymphohistiocytic infiltrates without significant differences.

Data are presented in box and whiskers plots (min-max) with mean and all data points. Perivascular lymphohistiocytic infiltrates (➡). Bars (A1-C2) = 50 µm. Hematoxylin and eosin staining (HE).

Supplementary Fig. 3: T cell infiltration in the hippocampus

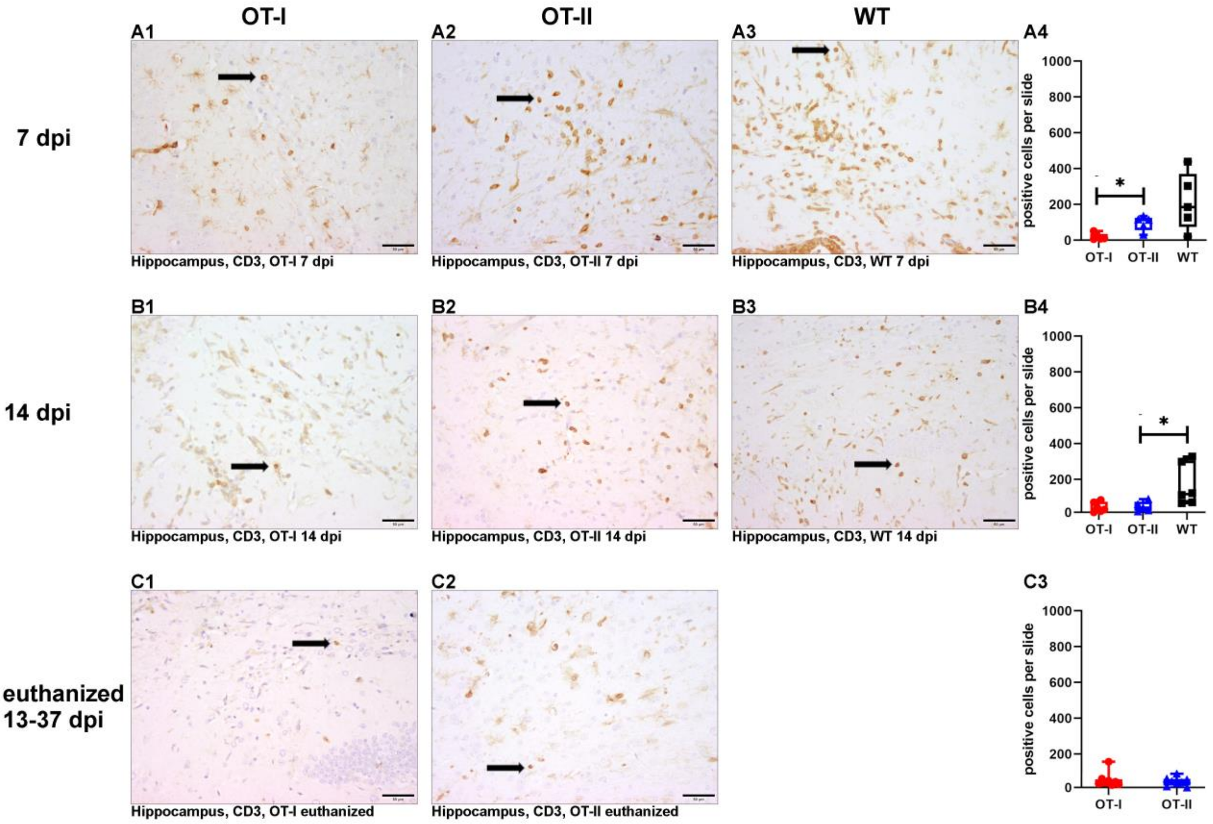

Suppl. Fig. 3: CD3$^+$ T cell infiltration in the hippocampus

Total numbers of CD3$^+$ T cells (arrows) in the hippocampus of Theiler's murine encephalomyelitis virus (TMEV) infected OT-I and OT-II mice as well as C57BL/6 wild type (WT) control mice.

A1-A4: At 7 dpi, OT-II mice displayed an increased CD3$^+$ T cell infiltration compared to OT-I mice (p=0.043) and no significant difference compared to WT mice.

B1-B4: At 14 dpi, WT mice displayed significantly increased CD3$^+$ T cell infiltrates compared to OT-II mice (p=0.046).

C1-C3: Animals euthanized for humane reasons at 13 - 37 dpi displayed a similar degree of CD3$^+$ T cell infiltrates without significant differences.

Data are presented in box and whiskers plots (min-max) with mean and all data points. T cells (➡). Bars (A1-C2) = 50 µm. ABC-DAB-immunohistochemistry, CD3, polyclonal.

Supplementary Fig. 4: T cell infiltration in the spinal cord

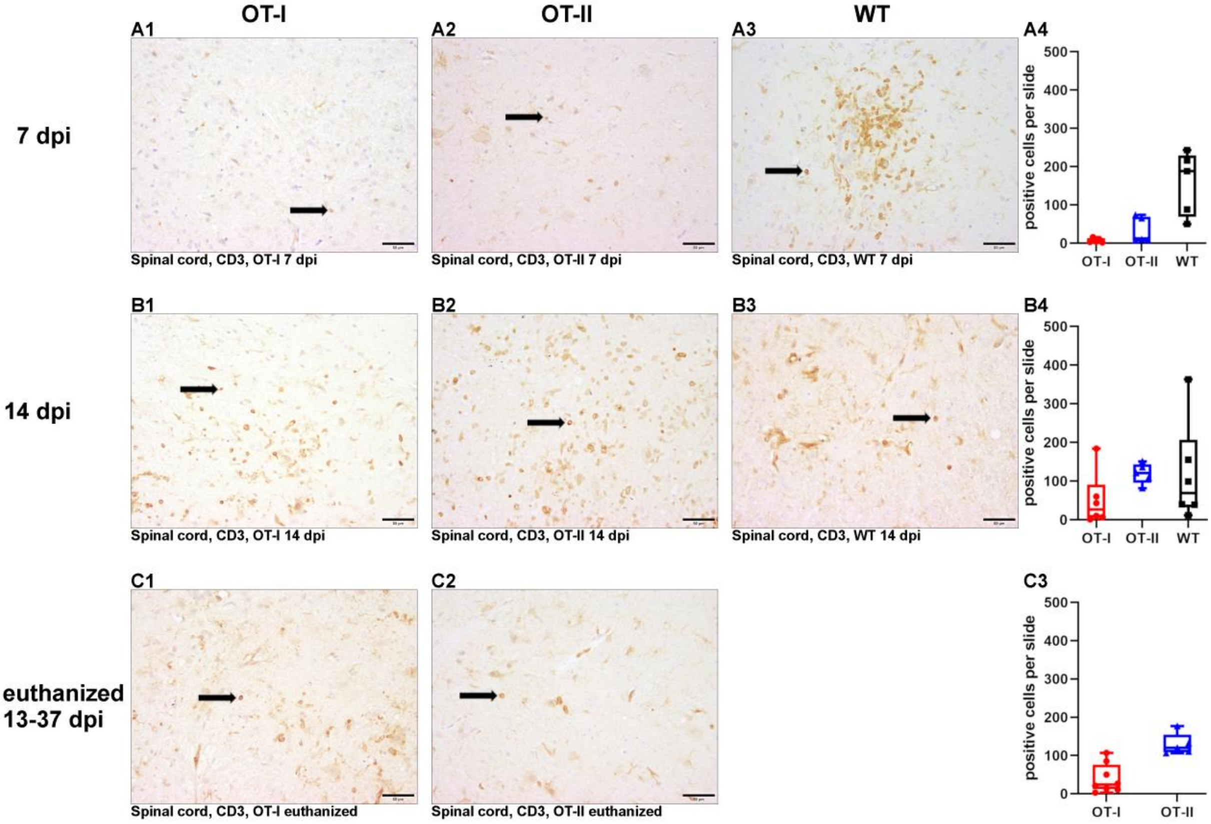

Suppl. Fig. 4: CD3$^+$ T cell infiltration in the spinal cord

Total numbers of CD3$^+$ T cells (arrows) in three spinal cord sections (cervical, thoracic, and lumbar) of Theiler's murine encephalomyelitis virus (TMEV) infected OT-I and OT-II mice as well as C57BL/6 wild type (WT) control mice.

A1-A4: At 7 dpi, WT mice displayed increased CD3$^+$ T cell infiltrates compared to OT-I mice (p=0.025).

B1-B4: At 14 dpi, all mice displayed a similar degree of CD3$^+$ T cell infiltrates without significant differences.

C1-C3: In animals euthanized for humane reasons at 13 - 37 dpi, OT-II mice displayed increased CD3$^+$ T cell infiltrates compared to OT-I mice (p=0.019).

Data are presented in box and whiskers plots (min-max) with mean and all data points. T cells (➡). Bars (A1-C2) = 50 µm. ABC-DAB-immunohistochemistry, CD3, polyclonal.

Supplementary Fig. 5: CD4$^+$ T cell infiltration in the hippocampus

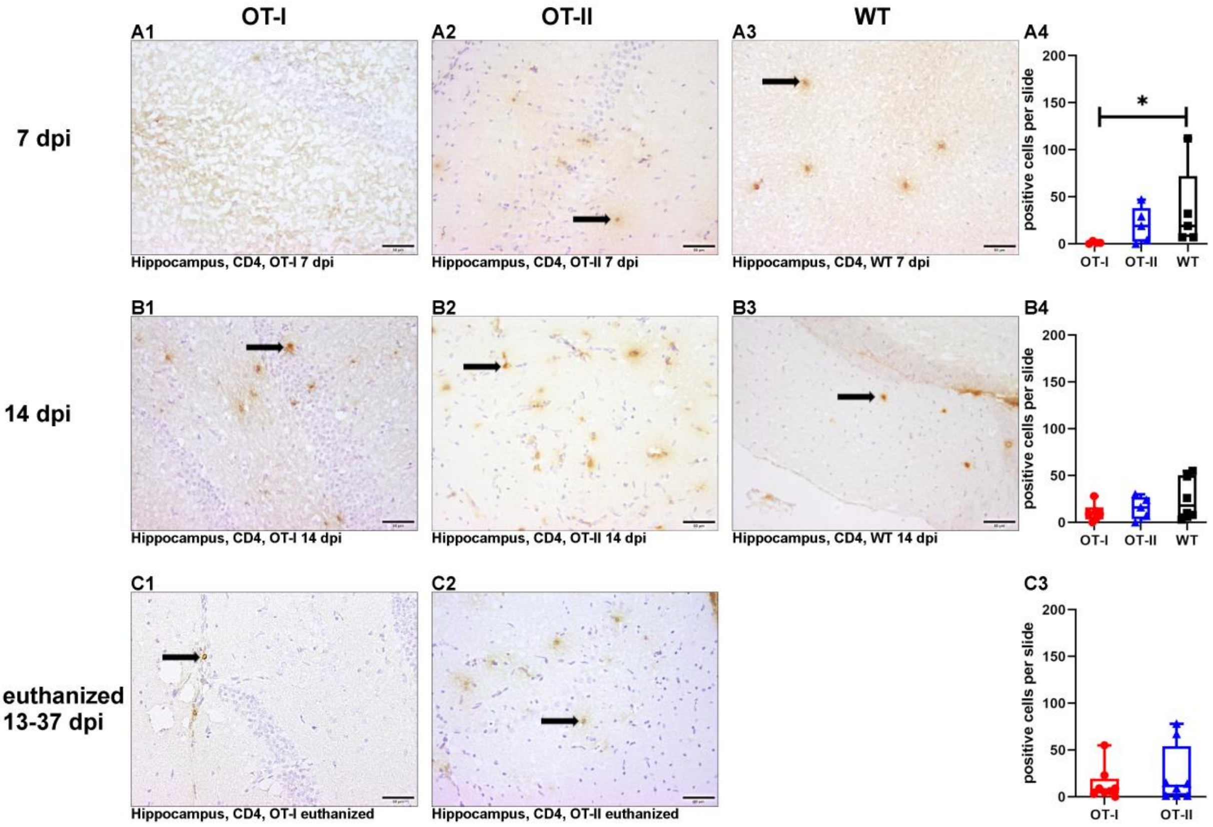

Suppl. Fig. 5: CD4$^+$ T cell infiltration in the hippocampus

Total numbers of CD4$^+$ T cells (arrows) in the hippocampus of Theiler's murine encephalomyelitis virus (TMEV) infected OT-I and OT-II mice as well as C57BL/6 wild type (WT) control mice.

A1-A4: At 7 dpi, WT mice displayed increased CD4$^+$ T cell infiltrates compared to OT-I mice (p=0.036).

B1-B4: At 14 dpi, no significant differences were detected between the study groups with respect to CD4$^+$ T cell infiltrates.

C1-C3: Animals euthanized for humane reasons at 13 - 37 dpi displayed a similar degree of CD4$^+$ T cell infiltrates without significant differences.

Data are presented in box and whiskers plots (min-max) with mean and all data points. CD4$^+$ T cells (➡). Bars (A1-C2) = 50 µm. ABC-DAB-immunohistochemistry, CD4, monoclonal.

Supplementary Fig. 6: CD4$^+$ T cell infiltration in the spinal cord

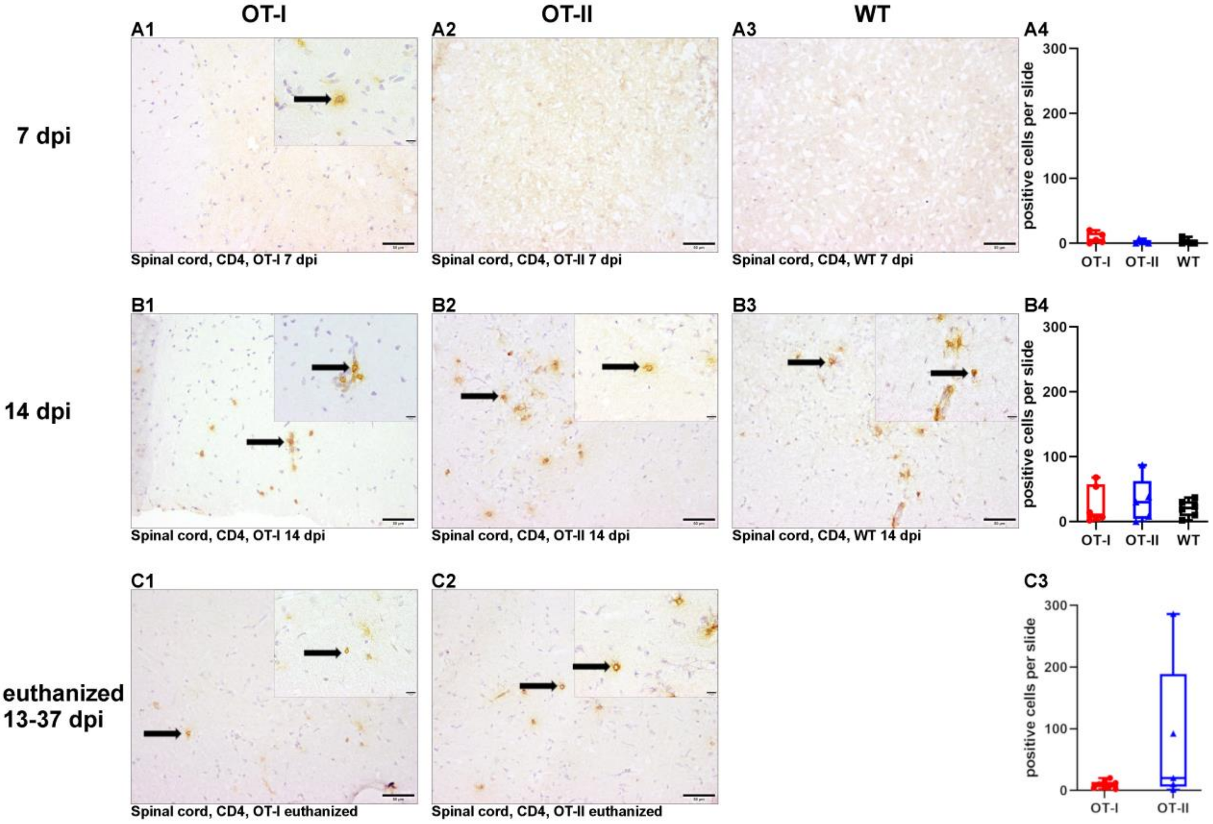

Suppl. Fig. 6: CD4$^+$ T cell infiltration in the spinal cord

Total numbers of CD4$^+$ T cells (arrows) in three spinal cord sections (cervical, thoracic, and lumbar) of Theiler's murine encephalomyelitis virus (TMEV) infected OT-I and OT-II mice as well as C57BL/6 wild type (WT) control mice.

A1-A4: At 7 dpi, no significant differences were detected between the study groups with respect to CD4+ T cell infiltrates.

B1-B4: At 14 dpi, no significant differences were detected between the study groups with respect to CD4+ T cell infiltrates.

C1-C3: Animals euthanized for humane reasons at 13 - 37 dpi displayed a similar degree of CD4$^+$ T cell infiltrates without significant differences.

Data are presented in box and whiskers plots (min-max) with mean and all data points. CD4$^+$ T cells (➡). Bars (A1-C2) = 50 µm. Bars in inserts (A1, B1-C2) = 10 µm. ABC-DAB-immunohistochemistry, CD4, monoclonal.

Supplementary Fig. 7: CD8^{+} T cell infiltration in the hippocampus

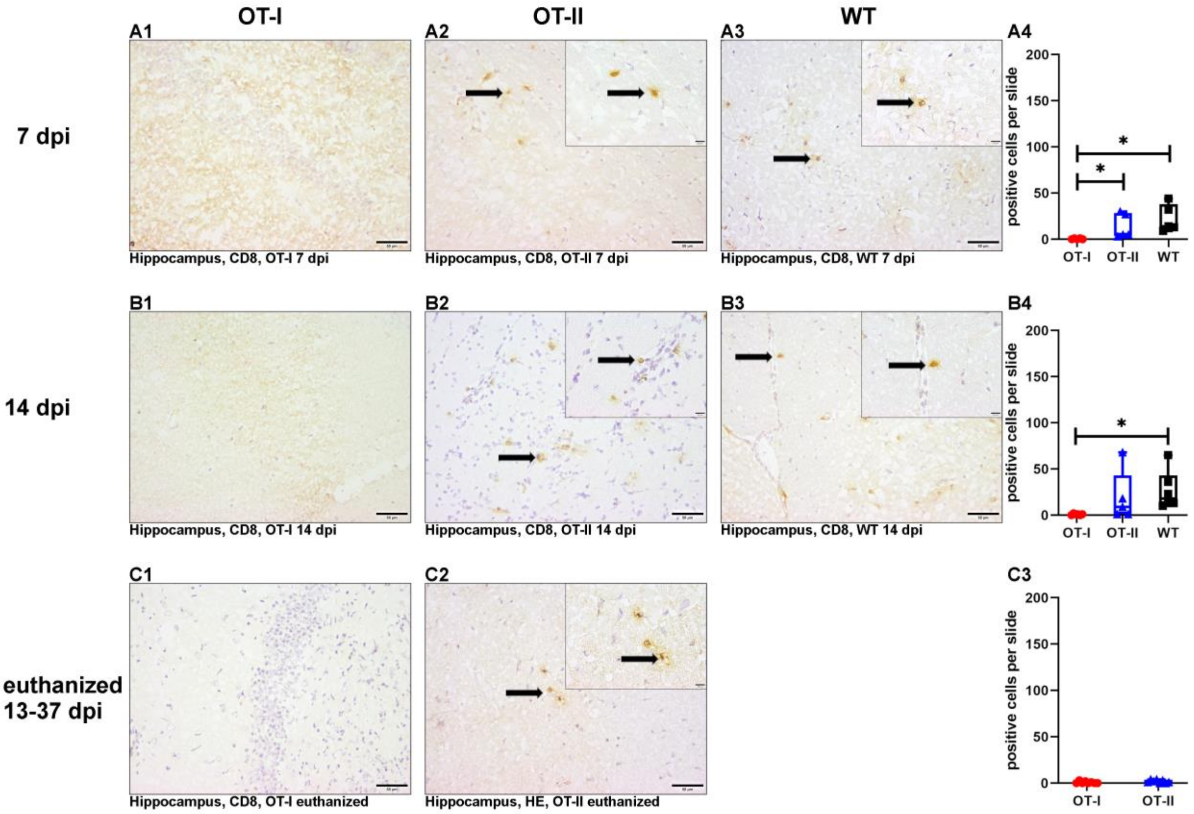

Suppl. Fig. 7: CD8^{+} T cell infiltration in the hippocampus

Total numbers of CD8^{+} T cells (arrows) in the hippocampus of Theiler's murine encephalomyelitis virus (TMEV) infected OT-I and OT-II mice as well as C57BL/6 wild type (WT) control mice.

A1-A4: At 7 dpi, OT-II and WT mice displayed increased CD8^{+} T cell infiltrates compared to OT-I mice (OT-I vs. OT-II p=0.021, OT-I vs. WT p=0.022).

B1-B4: At 14 dpi, WT mice still displayed increased CD8^{+} T cell infiltrates compared to OT-I mice (p=0.01), but not OT-II mice.

C1-C3: Animals euthanized for humane reasons at 13 - 37 dpi displayed a similar degree of CD8^{+} T cell infiltration without significant differences.

Data are presented in box and whiskers plots (min-max) with mean and all data points. CD8^{+} T cells (➡). Bars (A1-C2) = 50 µm. Bars in inserts (A2-3,B2-3, C2) = 10 µm. ABC- DAB- immunohistochemistry, CD8, monoclonal.

Supplementary Fig. 8: CD8⁺ T cell infiltration in the spinal cord

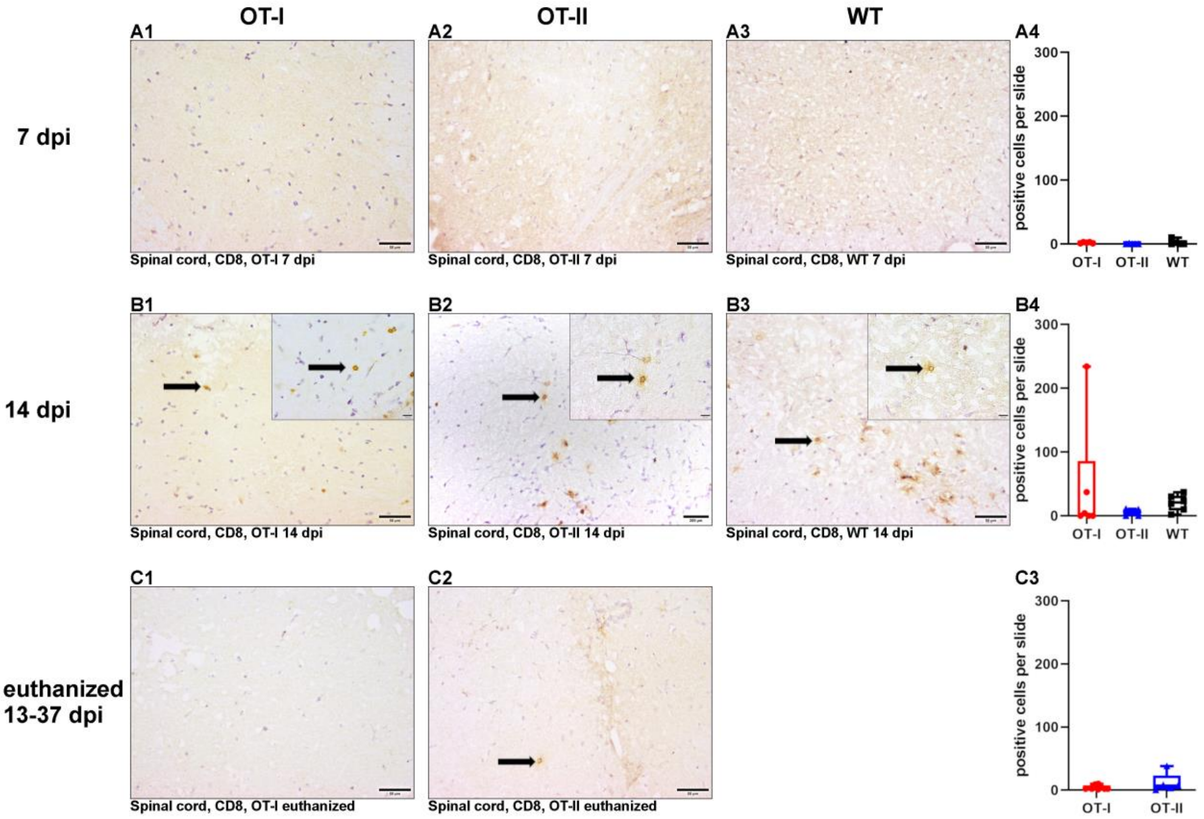

Suppl. Fig. 8: CD8$^+$ T cell infiltration in the spinal cord
Total numbers of CD8$^+$ T cells (arrows) in three spinal cord sections (cervical, thoracic, and lumbar) of Theiler's murine encephalomyelitis virus (TMEV) infected OT-I and OT-II mice as well as C57BL/6 wild type (WT) control mice.

A1-A4: At 7 dpi, no significant difference was detected in CD8$^+$ T cell infiltrates between the study groups.

B1-B4: At 14 dpi, no significant difference was detected in CD8$^+$ T cell infiltrates between the study groups.

C1-C3: Animals euthanized for humane reasons at 13 - 37 dpi displayed similar numbers of infiltrating CD8$^+$ T cells within the spinal cord without significant differences.

Data are presented in box and whiskers plots (min-max) with mean and all data points. CD8$^+$ T cells (➡). Bars (A1-C2) = 50 µm. Bars in inserts (B1-3) = 10 µm. ABC- DAB-immunohistochemistry, CD8, monoclonal.

Supplementary Fig. 9: TMEV immunoreactivity in the spinal cord

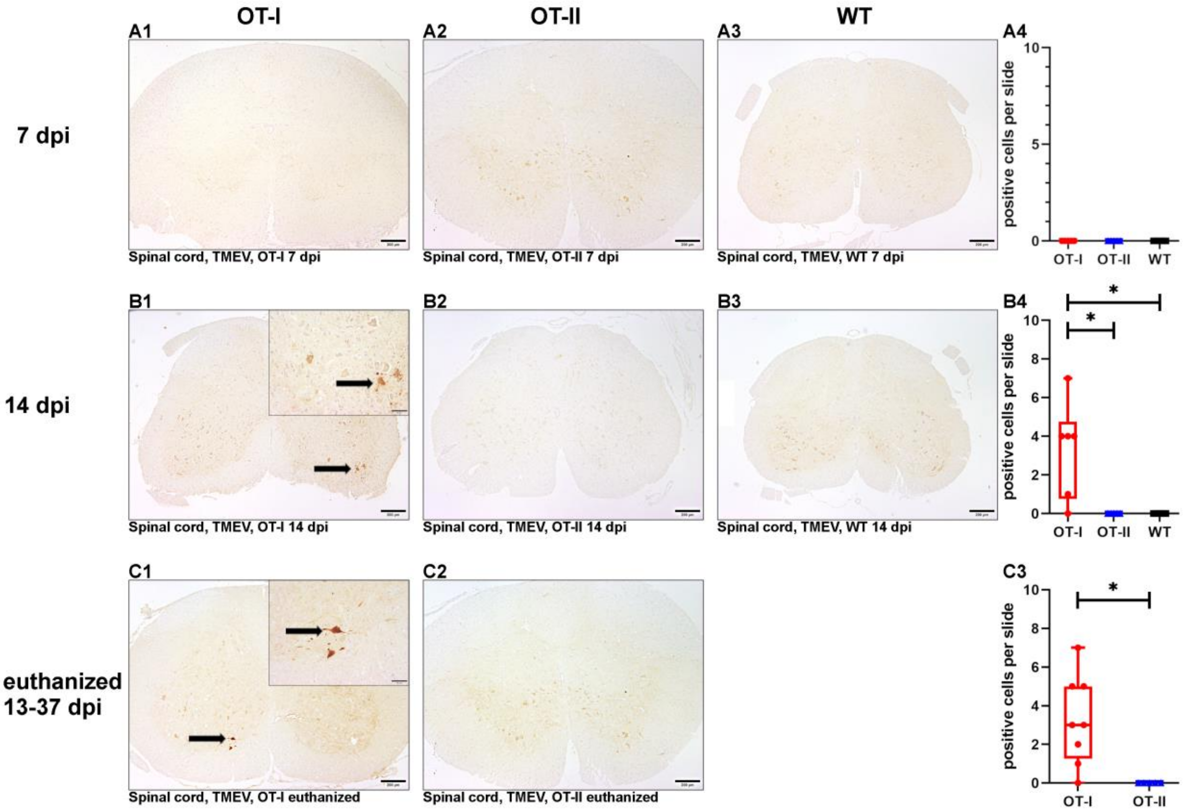

Suppl. Fig. 9: TMEV immunoreactivity in the spinal cord
Total numbers of Theiler's murine encephalomyelitis virus (TMEV) antigen-positive cells (arrows) in three spinal cord sections (cervical, thoracic, and lumbar) of TMEV-infected OT-I and OT-II mice as well as C57BL/6 wild type (WT) control mice. Inserts display a higher magnification.

A1-A4: At 7 dpi, no viral antigen was detected in the spinal cords of all study groups.

B1-B4: At 14 dpi, OT-I mice displayed virus spread to the spinal cord, while no viral antigen was detected in the spinal cord of OT-II and WT mice (OT-I vs. WT p=0.019, OT-I vs. OT-II p=0.032).

C1-C3: In animals euthanized for humane reasons at 13 - 37 dpi, OT-I mice displayed continuously viral antigen in the spinal cord, while no viral antigen was detected in OT-II mice (p=0.004).

Data are presented in box and whiskers plots (min-max) with mean and all data points. TMEV-positive cells (➡). Bars (A1-C2) = 200 µm. Bars in inserts (B1,C1) = 10 µm. ABC- DAB- immunohistochemistry, Theiler's murine encephalomyelitis virus (TMEV), polyclonal.

Supplementary Fig. 10: TMEV immunoreactivity in the hippocampus

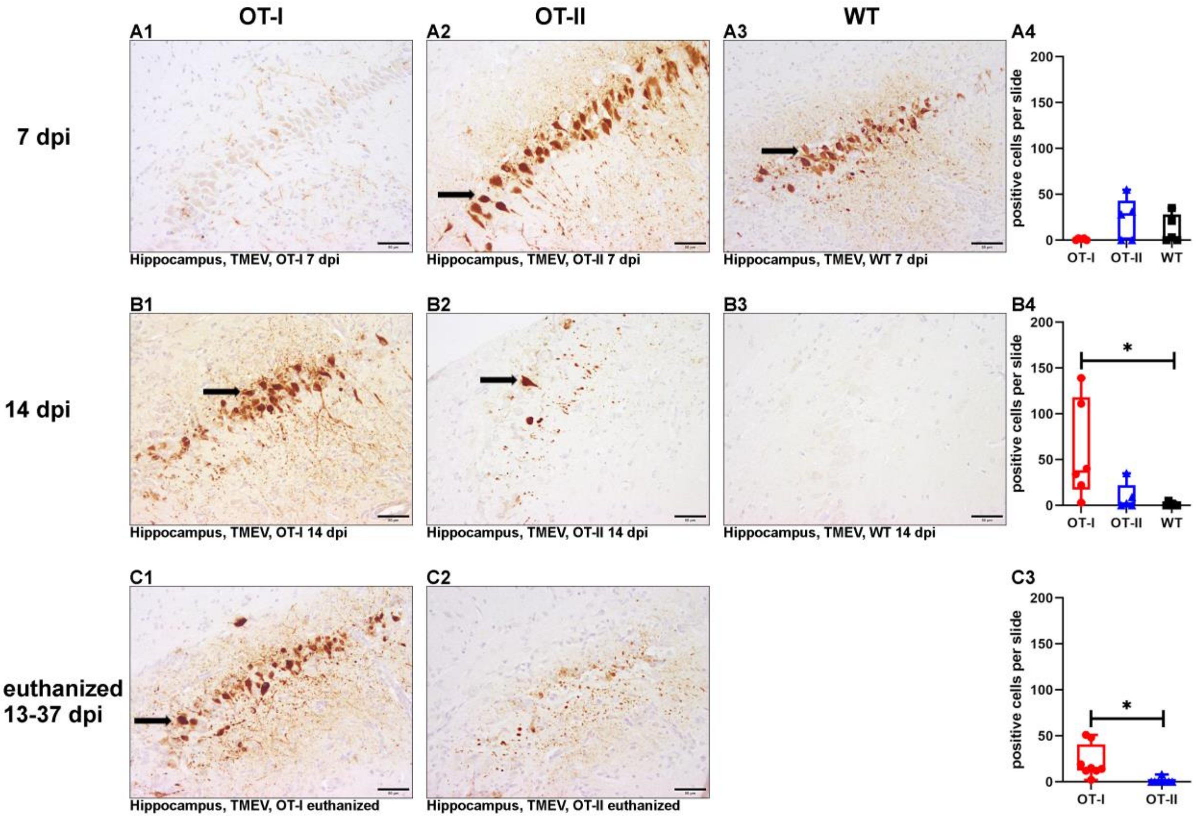

Suppl. Fig. 10: TMEV-immunoreactivity in the hippocampus

Total numbers of Theiler's murine encephalomyelitis virus (TMEV) antigen-positive cells (arrows) in the hippocampus of TMEV-infected OT-I and OT-II mice as well as C57BL/6 wild type (WT) control mice.

A1-A4: At 7 dpi, no significant difference in virus antigen immunoreactivity was detected between all study groups. Despite variable expression patterns, the statistical analysis revealed no significant difference within the study groups.

B1-B4: At 14 dpi, OT-I mice displayed increased numbers of virus antigen-positive cells compared to WT mice (p=0.015).

C1-C3: In animals euthanized for humane reasons at 13 - 37 dpi, OT-I mice displayed an increased number of virus antigen-positive cells compared to OT-II mice (p=0.018).

Data are presented in box and whiskers plots (min-max) with mean and all data points. TMEV-positive cells (➡). Bars (A1-C2) = 50 µm. ABC-DAB-immunohistochemistry, Theiler's murine encephalomyelitis virus (TMEV), polyclonal.

Supplementary Fig. 11: Microglia/macrophages in the hippocampus

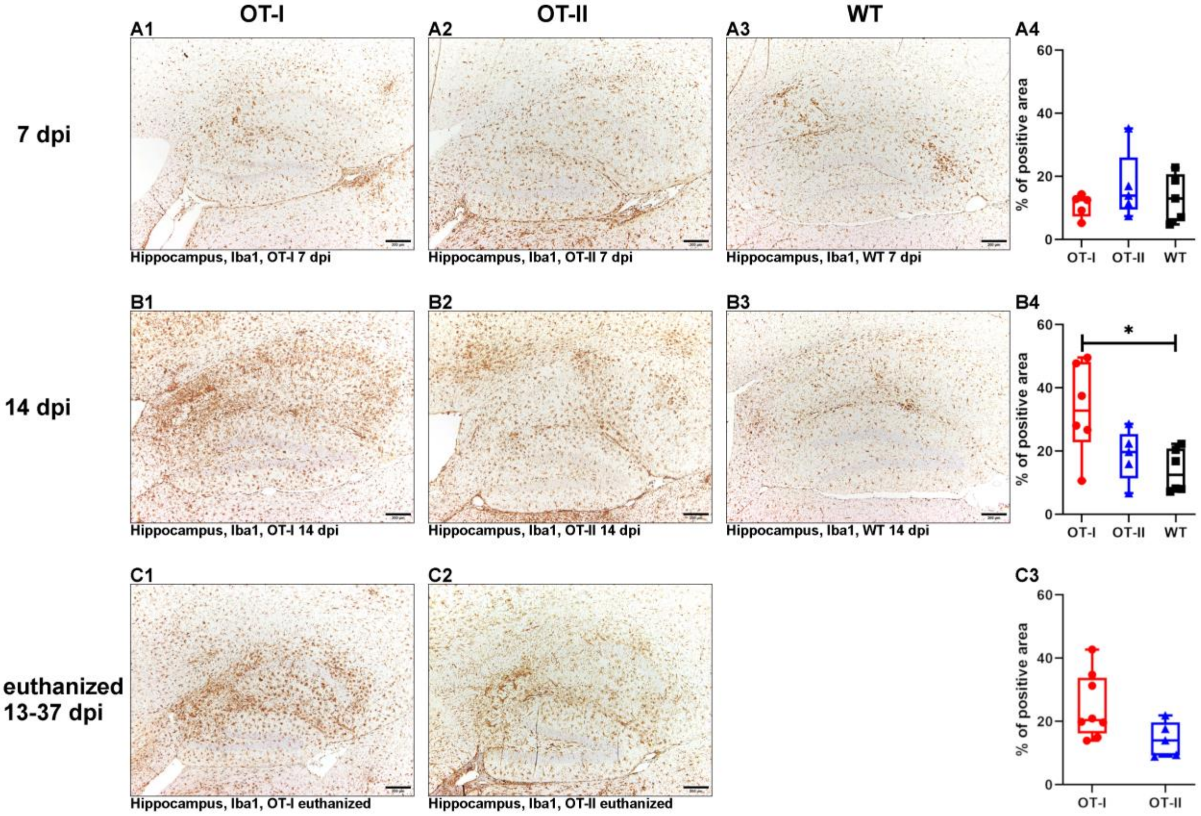

Suppl. Fig. 11: Microgliosis and macrophage infiltration in the hippocampus measured by detection of Iba1

Percent (%) of area occupied by Iba1 labelled cells in the hippocampus of Theiler's murine encephalomyelitis virus (TMEV) infected OT-I and OT-II mice as well as C57BL/6 wild type (WT) control mice.

A1-A4: At 7 dpi, no significant difference in microglia/macrophage infiltration was detected between all study groups.

B1-B4: At 14 dpi, OT-I mice displayed an increased infiltration of microglia/macrophages compared to WT mice (p=0.043).

C1-C3: Animals euthanized for humane reasons at 13 - 37 dpi displayed no significant difference in microglia/macrophage infiltration.

Data are presented in box and whiskers plots (min-max) with mean and all data points. Bars (A1-C2) = 200 µm. ABC- DAB- immunohistochemistry, ionized calcium-binding adapter molecule 1 (Iba1), polyclonal.

Supplementary Fig. 12: Microglia/macrophages in the spinal cord

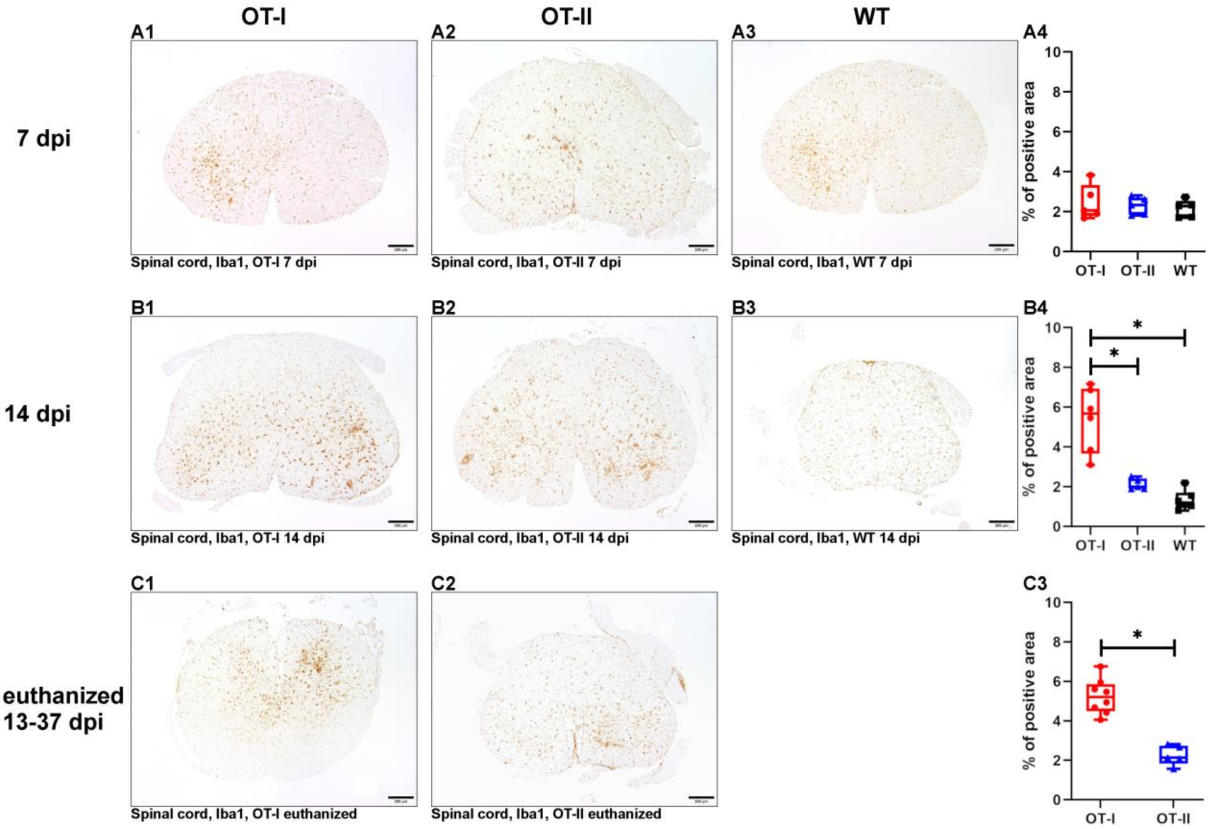

Suppl. Fig. 12: Microgliosis and macrophage infiltration in the spinal cord measured by detection of Iba1

Percent (%) of area occupied by Iba1 labelled cells in three spinal cord sections (cervical, thoracic, and lumbar) of Theiler's murine encephalomyelitis virus (TMEV) infected OT-I and OT-II mice as well as C57BL/6 wild type (WT) control mice.

A1-A4: At 7 dpi, no significant difference in microglia/macrophage infiltration was detected between the study groups.

B1-B4: At 14 dpi, OT-I mice displayed an increased microglia/macrophage compared to OT-II and WT mice (OT-I vs. OT-II p=0.017, OT-I vs. WT p=0.011).

C1-C3: In animals euthanized for humane reasons at 13 - 37 dpi, OT-I mice displayed an increased spinal cord microgliosis compared to OT-II mice (p=0.015).

Data are presented in box and whiskers plots (min-max) with mean and all data points. Microglia (➡). Bars (A1-C2) = 200 µm. ABC- DAB- immunohistochemistry, ionized calcium-binding adapter molecule 1 (Iba1), polyclonal.

Supplementary Fig. 13: Neuronal apoptosis in the hippocampus

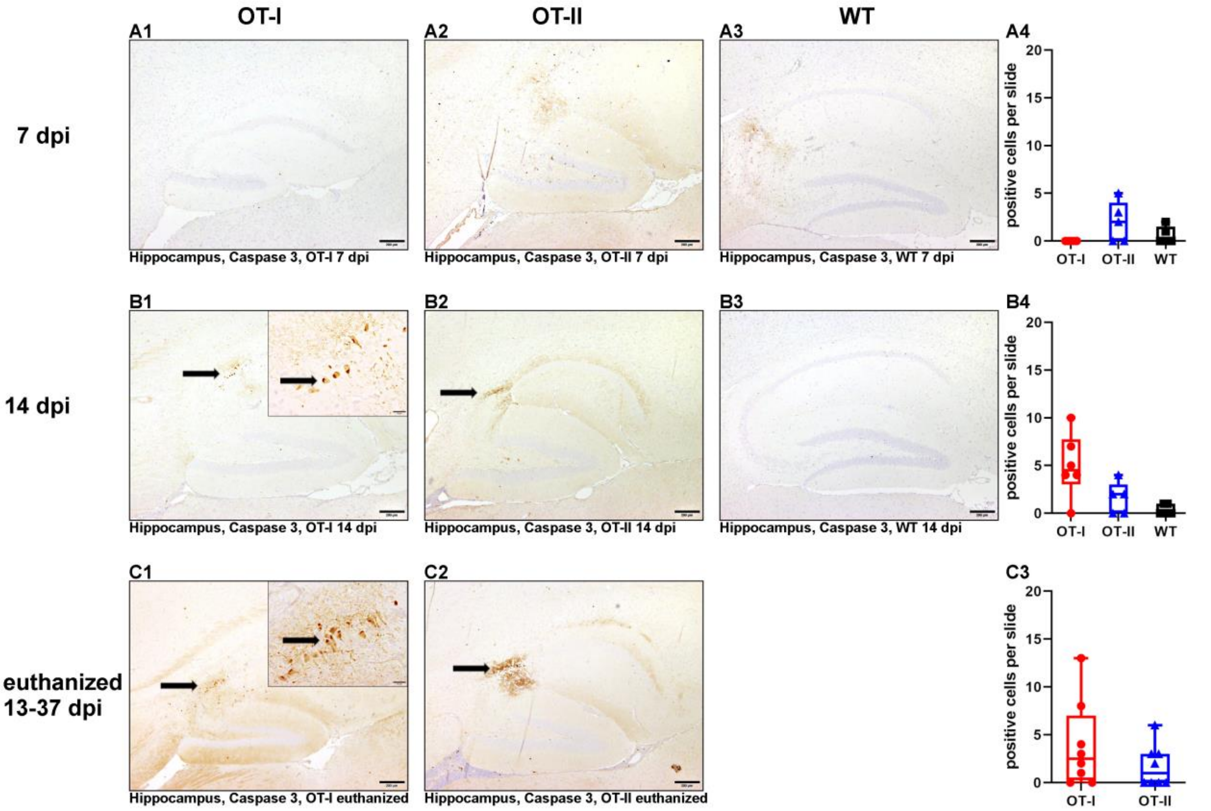

Suppl. Fig. 13: Neuronal apoptosis in hippocampus measured by positive cleaved caspase 3 signalling

Total numbers of cleaved caspase 3- positive, apoptotic cells (arrows) in the hippocampus of Theiler's murine encephalomyelitis virus (TMEV) infected OT-I and OT-II mice as well as C57BL/6 wild type (WT) control mice. Inserts display a higher magnification.

A1-A4: At 7 dpi, no significant difference in neuronal apoptosis was detected between all study groups.

B1-B4: At 14 dpi, no significant difference in neuronal apoptosis was detected between all study groups.

C1-C3: Animals euthanized for humane reasons at 13 - 37 dpi displayed no significant difference in neuronal apoptosis.

Data are presented in box and whiskers plots (min-max) with mean and all data points. Cleaved caspase 3- positive neurons (➡). Bars (A1-C2) = 200 μm. Bars in inserts (B1,C1) = 50 μm. ABC- DAB- immunohistochemistry, cleaved caspase 3, polyclonal.

Supplementary Fig. 14: Neuronal apoptosis in the spinal cord

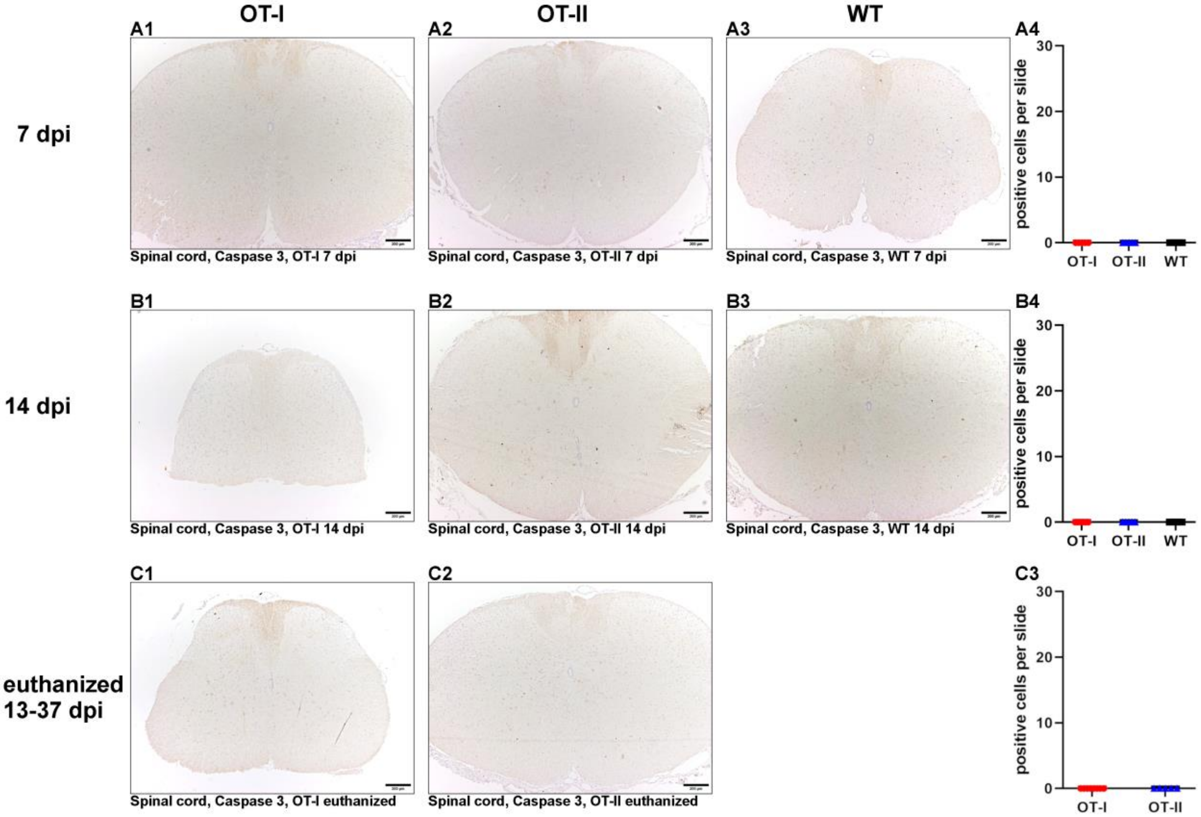

Suppl. Fig. 14: Neuronal apoptosis in the spinal cord measured by cleaved caspase 3 signalling

Total numbers of cleaved caspase 3-positive, apoptotic cells (arrows) in three spinal cord sections (cervical, thoracic, and lumbar) of Theiler's murine encephalomyelitis virus (TMEV) infected OT-I and OT-II mice as well as C57BL/6 wild type (WT) control mice.

A1-A4: At 7 dpi, no neuronal apoptosis was detected in all study groups.

B1-B4: At 14 dpi, no neuronal apoptosis was detected in all study groups.

C1-C3: Animals euthanized for humane reasons at 13 - 37 dpi displayed no neuronal apoptosis.

Data are presented in box and whiskers plots (min-max) with mean and all data points. Bars (A1-C2) = 200 µm. ABC-DAB-immunohistochemistry, cleaved caspase 3, polyclonal.

Supplementary Fig. 15: GFAP-positive cells in the hippocampus

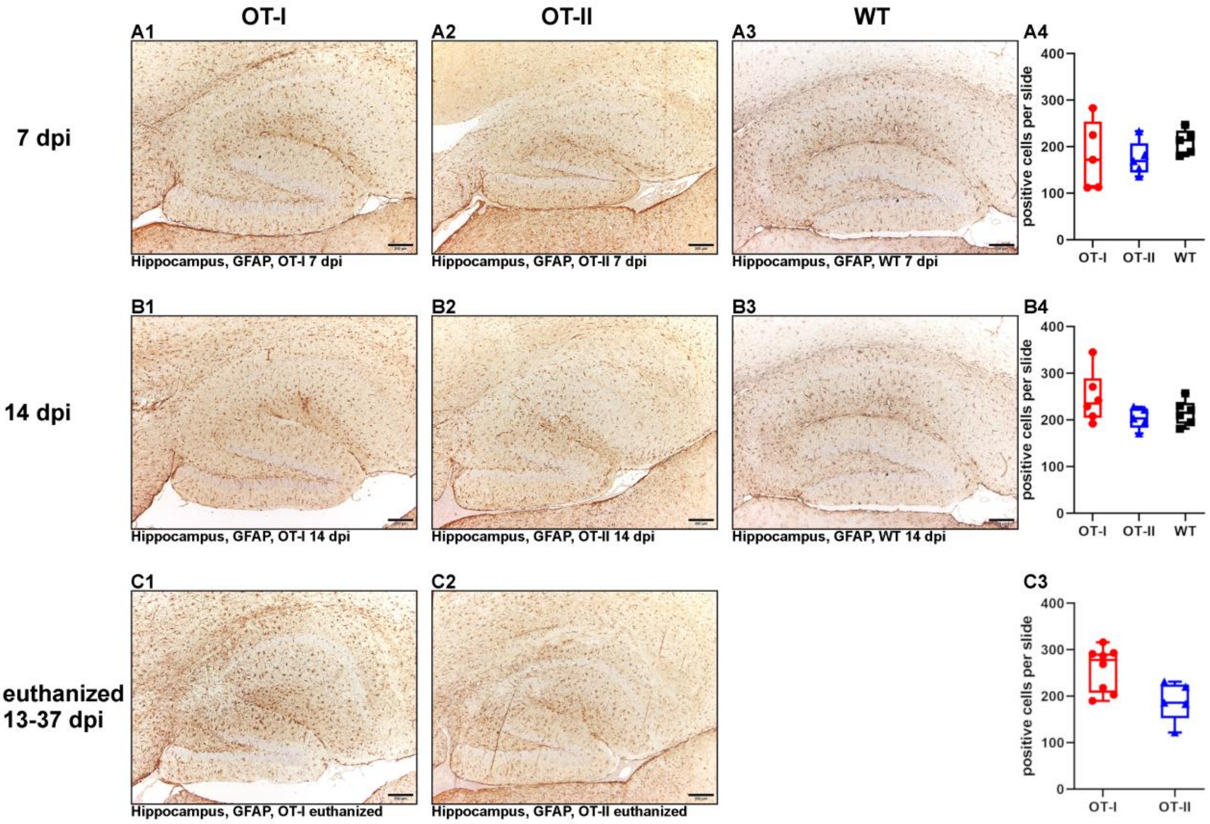

Suppl. Fig. 15: Glial fibrillary acidic protein (GFAP) - positive cells in the hippocampus Total numbers of GFAP-positive cells (arrows) in the hippocampus of Theiler's murine encephalomyelitis virus (TMEV) infected OT-I and OT-II mice as well as C57BL/6 wild type (WT) control mice.

A1-A4: At 7 dpi, all study groups displayed similar numbers of glial fibrillary acidic protein - positive cells without significant differences.

B1-B4: At 14 dpi, all study groups also displayed similar numbers of glial fibrillary acidic protein - positive cells without significant differences.

C1-C3: Animals euthanized for humane reasons at 13 - 37 dpi displayed similar numbers of glial fibrillary acidic protein - positive cells without significant differences.

Data are presented in box and whiskers plots (min-max) with mean and all data points. Bars (A1-C2) = 200 µm. ABC-DAB-immunohistochemistry, glial fibrillary acidic protein (GFAP), polyclonal.

Supplementary Fig. 16: Neuronal NeuN expression in the spinal cord

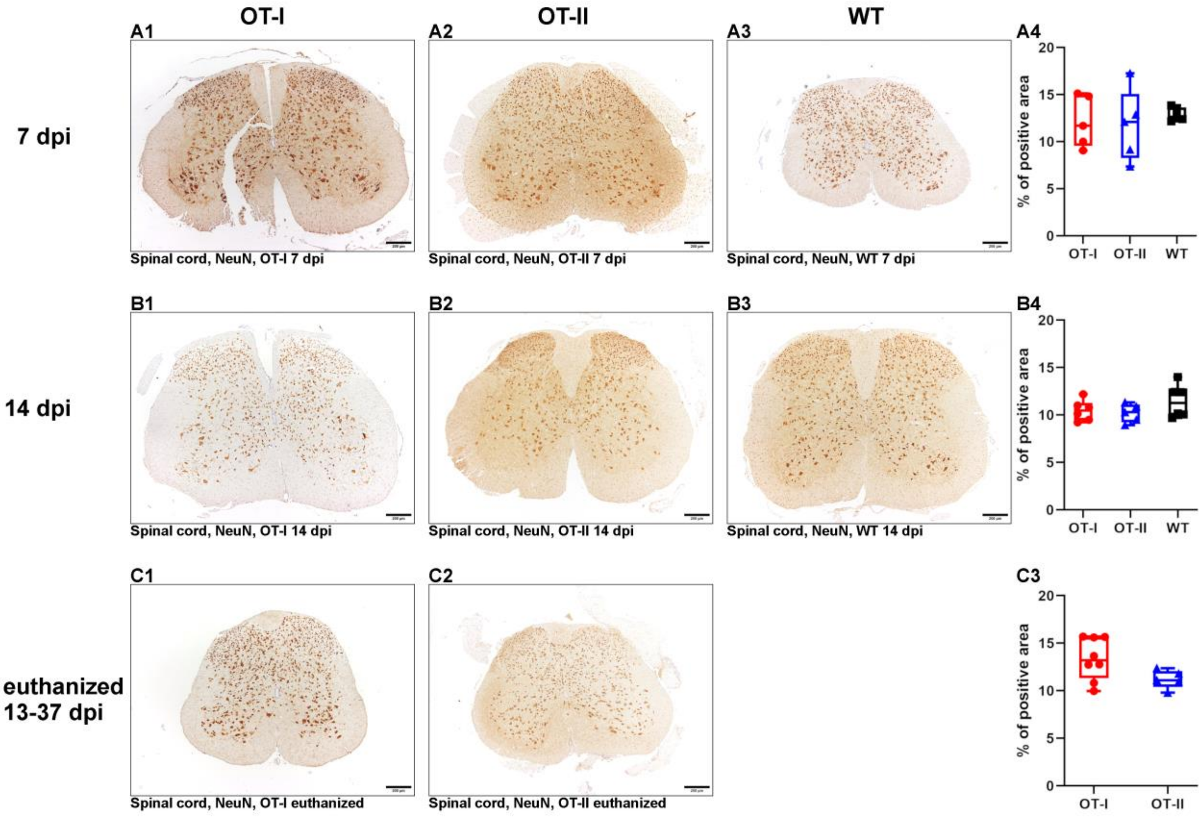

Suppl. Fig. 16: relative area of NeuN expression in the spinal cord

Percent (%) of area occupied by NeuN- positive neurons in three spinal cord sections (cervical, thoracic, and lumbar) of Theiler's murine encephalomyelitis virus (TMEV) infected OT-I and OT-II mice as well as C57BL/6 wild type (WT) control mice.

A1-A4: At 7 dpi, all study groups displayed a similar pattern of NeuN expression in the spinal cord without significant differences.

B1-B4: At 14 dpi, all study groups displayed a similar pattern of NeuN expression in the spinal cord without significant differences.

C1-C3: in animals euthanized for humane reasons at 13 - 37 dpi, OT-I and OT-II mice displayed a similar pattern of NeuN expression in the spinal cord without significant differences.

Bars (A1-C2) = 200 µm. ABC-DAB-immunohistochemistry, neuronal nuclear protein (NeuN), monoclonal.

Supplementary Fig. 17: Axonal damage in the hippocampus

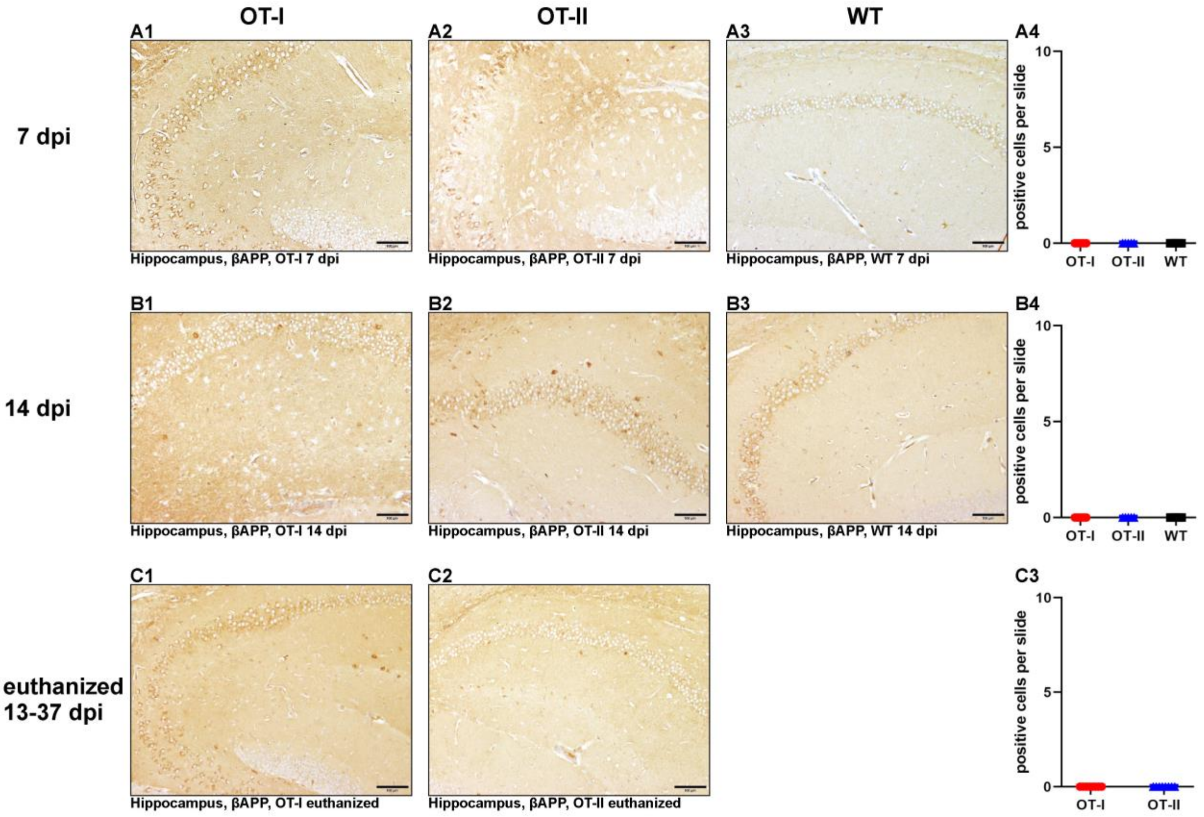

Suppl. Fig. 17: Axonal damage in the hippocampus, measured by axonal detection of β-amyloid precursor protein (βAPP)

Total numbers of βAPP-positive axons in the hippocampus of Theiler's murine encephalomyelitis virus (TMEV) infected OT-I and OT-II mice as well as C57BL/6 wild type (WT) control mice.

A1-A4: At 7 dpi, no study group displayed axonal damage in the hippocampus.

B1-B4: At 14 dpi, no study group displayed axonal damage in the hippocampus.

C1-C3: In animals euthanized for humane reasons at 13-37 dpi, no study group displayed axonal damage in the hippocampus.

Data are presented in box and whiskers plots (min-max) with mean and all data points. Bars (A1-C2) = 50 µm. ABC-DAB-immunohistochemistry, β- amyloid precursor protein (βAPP), monoclonal.

Supplementary Fig. 18: Axonal damage in the brain

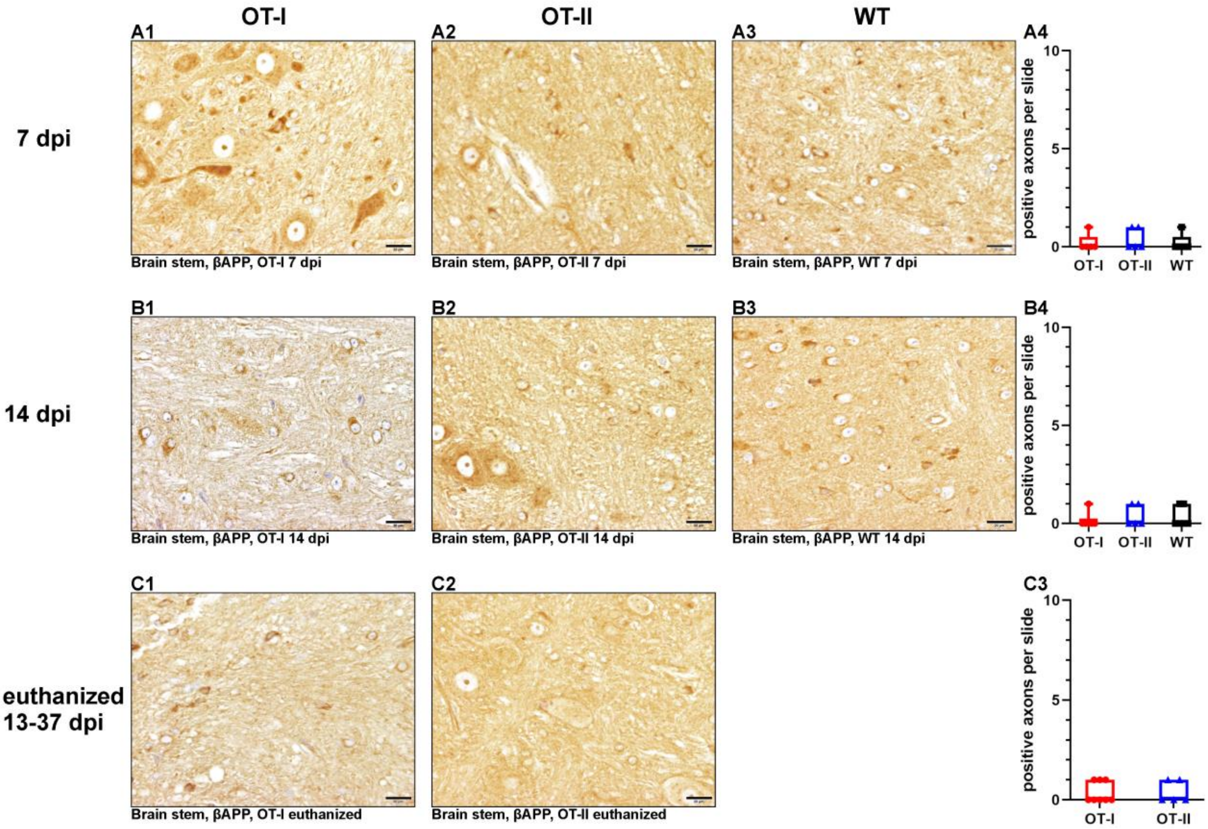

Suppl. Fig. 18: Cerebral axonal damage, measured by axonal detection of β-amyloid precursor protein (βAPP)

Total numbers of βAPP-positive axons in the brain of Theiler's murine encephalomyelitis virus (TMEV) infected OT-I and OT-II mice as well as C57BL/6 wild type (WT) control mice.

A1-A4: At 7 dpi, no study groups displayed axonal damage in the brain.

B1-B4: At 14 dpi, no study groups displayed axonal damage in the brain.

C1-C3: In animals euthanized for humane reasons at 13-37 dpi, no study groups displayed axonal damage in the brain.

Data are presented in box and whiskers plots (min-max) with mean and all data points. Bars (A1-C2) = 50 µm. ABC-DAB- immunohistochemistry, β-amyloid precursor protein (βAPP), monoclonal.

Supplementary Fig. 19: Axonal damage in the spinal cord

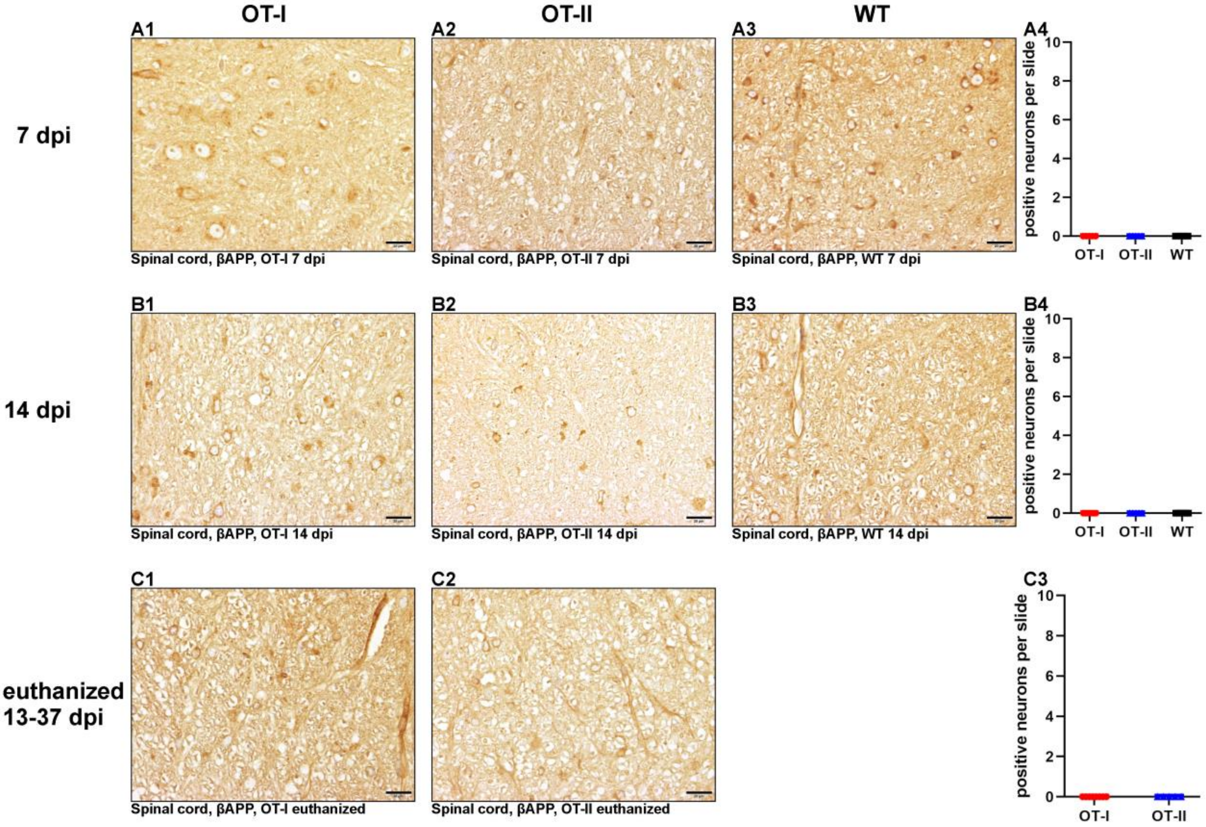

Suppl. Fig. 19: Axonal damage in the spinal cord, measured by axonal detection of β-amyloid precursor protein (βAPP)

Total numbers of βAPP-positive axons in three spinal cord sections (cervical, thoracic, and lumbar) of Theiler's murine encephalomyelitis virus (TMEV) infected OT-I and OT-II mice as well as C57BL/6 wild type (WT) control mice.

A1-A4: At 7 dpi, no study group displayed axonal damage in the spinal cord.

B1-B4: At 14 dpi, no study group displayed axonal damage in the spinal cord.

C1-C3: In animals euthanized for humane reasons at 13-37 dpi, no study group displayed axonal damage in the spinal cord.

Data are presented in box and whiskers plots (min-max) with mean and all data points. Bars (A1-C2) = 50 µm. ABC-DAB-immunohistochemistry, β-amyloid precursor protein (βAPP), monoclonal.
